# Dietary zinc and the control of *Streptococcus pneumoniae* infection

**DOI:** 10.1371/journal.ppat.1007957

**Published:** 2019-08-22

**Authors:** Bart A. Eijkelkamp, Jacqueline R. Morey, Stephanie L. Neville, Aimee Tan, Victoria G. Pederick, Nerida Cole, Prashina P. Singh, Cheryl-Lynn Y. Ong, Raquel Gonzalez de Vega, David Clases, Bliss A. Cunningham, Catherine E. Hughes, Iain Comerford, Erin B. Brazel, Jonathan J. Whittall, Charles D. Plumptre, Shaun R. McColl, James C. Paton, Alastair G. McEwan, Philip A. Doble, Christopher A. McDevitt

**Affiliations:** 1 Research Centre for Infectious Diseases, Department of Molecular and Biomedical Science, School of Biological Sciences, The University of Adelaide, Adelaide, Australia; 2 Department of Molecular and Biomedical Science, School of Biological Sciences, The University of Adelaide, Adelaide, Australia; 3 Department of Microbiology and Immunology, The Peter Doherty Institute for Infection and Immunity, University of Melbourne, Melbourne, Victoria, Australia; 4 The Atomic Medicine Initiative, University of Technology, Broadway, Sydney, New South Wales, Australia; 5 ARC Training Centre in Biodevices, Faculty of Science, Engineering and Technology, Swinburne University of Technology, Hawthorn, Victoria, Australia; 6 Australian Infectious Diseases Research Centre, School of Chemistry and Molecular Biosciences, University of Queensland, Brisbane, Queensland, Australia; The University of Alabama at Birmingham, UNITED STATES

## Abstract

Human zinc deficiency increases susceptibility to bacterial infection. Although zinc supplementation therapies can reduce the impact of disease, the molecular basis for protection remains unclear. *Streptococcus pneumoniae* is a major cause of bacterial pneumonia, which is prevalent in regions of zinc deficiency. We report that dietary zinc levels dictate the outcome of *S*. *pneumoniae* infection in a murine model. Dietary zinc restriction impacts murine tissue zinc levels with distribution post-infection altered, and *S*. *pneumoniae* virulence and infection enhanced. Although the activation and infiltration of murine phagocytic cells was not affected by zinc restriction, their efficacy of bacterial control was compromised. *S*. *pneumoniae* was shown to be highly sensitive to zinc intoxication, with this process impaired in zinc restricted mice and isolated phagocytic cells. Collectively, these data show how dietary zinc deficiency increases sensitivity to *S*. *pneumoniae* infection while revealing a role for zinc as a component of host antimicrobial defences.

## Author summary

Zinc deficiency affects one-third of the world’s population and is associated with an increased susceptibility to bacterial infection. Despite this, the molecular basis for how zinc deficiency compromises host control of infection remains to be understood. We show that dietary zinc deficiency impacts host tissue zinc abundances and its mobilization during infection by the major respiratory pathogen *Streptococcus pneumoniae*. Zinc acts as a direct antimicrobial against the pathogen, mobilized by phagocytic cells as a component of the innate immune response. Although immune activation and infiltration of phagocytic cells is unaffected by host zinc status, the lack of antimicrobial zinc compromises bacterial control in zinc deficient hosts. These findings highlight the importance of zinc sufficiency in resisting bacterial infection.

## Introduction

Zinc (Zn) is the second most abundant transition metal ion in humans and has crucial importance in immune function [1]. Although severe Zn deficiency is rare, mild to moderate Zn deficiency is estimated to affect one third of the world’s population [2]. The impact on human health accounts for ~1.4% of annual global mortalities, manifesting in a variety of adverse clinical outcomes including compromised immune defence and an increased susceptibility to infections [3–5]. Bacterial diseases associated with Zn deficiency are typically caused by respiratory or enteric pathogens, resulting in pneumonia or diarrhoea, respectively [6, 7]. Clinical trials of Zn supplementation therapies have shown that the morbidity and mortality of pneumonia and diarrhoea can be significantly reduced [7–10]. However, the efficacy of Zn supplementation strategies varies, highlighting the fact that the mechanism by which Zn contributes to resistance against bacterial infections remains unclear.

*Streptococcus pneumoniae* (pneumococcus) is a globally significant human pathogen and the leading causative agent of bacterial pneumonia [11–14]. Pneumococcal disease is preceded by asymptomatic colonization of the nasopharynx, which occurs early in life with carriage rates reaching up to 90% in young children [15, 16]. Control of pneumococcal disease has primarily been addressed by vaccines that target the polysaccharide capsule of the bacterium. However, there are more than 90 distinct capsule variants (serotypes) with current vaccines providing protection against only a limited subset of serotypes [17, 18]. Further impacting the efficacy of vaccine strategies, disease monitoring has shown that the vaccine-included serotypes are being replaced by non-vaccine serotypes, against which current vaccines are not protective, and have not significantly reduced carriage rates [19, 20]. In addition, clinical interventions are increasingly hampered by the rising rates of resistance in *S*. *pneumoniae* to frontline antibiotic treatments [21, 22]. Pneumococcal pneumonia is highly prevalent in regions of endemic Zn-deficiency with mortality rates reaching up to 12% in children under 5 years of age [23, 24]. Although it is known that resistance to pneumococcal infection is significantly impaired by Zn deficiency [25, 26], the molecular basis for the control of *S*. *pneumoniae* proliferation and disease by Zn remains unclear.

Recent studies have highlighted the importance of first row transition metal ions, such as Zn, in host control of infection [25–28]. Vertebrate hosts have a diverse array of mechanisms to restrict metal ion availability, processes collectively referred to as nutritional immunity [29], which limit microbial infection. Despite this, pathogenic bacteria can overcome host metal ion sequestration by using metal-recruiting proteins and metallophores [30]. *S*. *pneumoniae* employs a combination of ATP-binding cassette (ABC) permeases to facilitate the acquisition of essential manganese (Mn), iron (Fe) and Zn ions from the host environment [31–34]. Zinc uptake is facilitated by the Adc permease, which comprises the ABC transporter AdcCB and two extra-cytoplasmic Zn recruiting proteins, AdcA and AdcAII [32, 35–39]. The extra-cytoplasmic protein components, also referred to as solute binding proteins (SBPs), enable scavenging of Zn ions from the mammalian host environment with active import occurring via the AdcCB transporter. This pathway is essential for Zn uptake as Adc permease deficient mutants have been shown to be attenuated in murine models of pneumococcal infection [32, 37]. Zn homeostasis in the pneumococcus is tightly regulated and sensed by two independent systems: the MarR-family regulator AdcR, which regulates expression of the *adc* permease and associated Zn-scavenging genes [40, 41]; and the TetR-family regulator SczA, which regulates expression of the Zn-efflux pathway gene *czcD* [42]. The latter system is also implicated in bacterial survival in the host, with recent studies highlighting the contribution of Zn intoxication in microbial clearance [43].

Here, we investigated host Zn status and how it influences susceptibility to pneumococcal infection. By combining dietary Zn restriction with a murine model of pneumococcal disease, we determined host Zn abundance and how this changes in clinically relevant niches during the progression of infection. Gene transcription profiling revealed that host niche Zn levels were directly sensed by the invading bacteria resulting in changes to metal ion homeostatic processes in the pathogen. Activation and infiltration of immune cells in response to pneumococcal infection was also analysed, in combination with *in vitro* and *ex vivo* assessment of phagocytic cells and how Zn influences their ability to kill *S*. *pneumoniae*. This study reveals the link between dietary Zn intake and host resistance to bacterial pneumonia, demonstrating the antimicrobial activity of Zn in host niches against invading *S*. *pneumoniae* and in potentiating the efficacy of phagocytic cell killing of the pathogen.

## Results

### The effect of diet on murine zinc and susceptibility to infection

**Fig 1:**
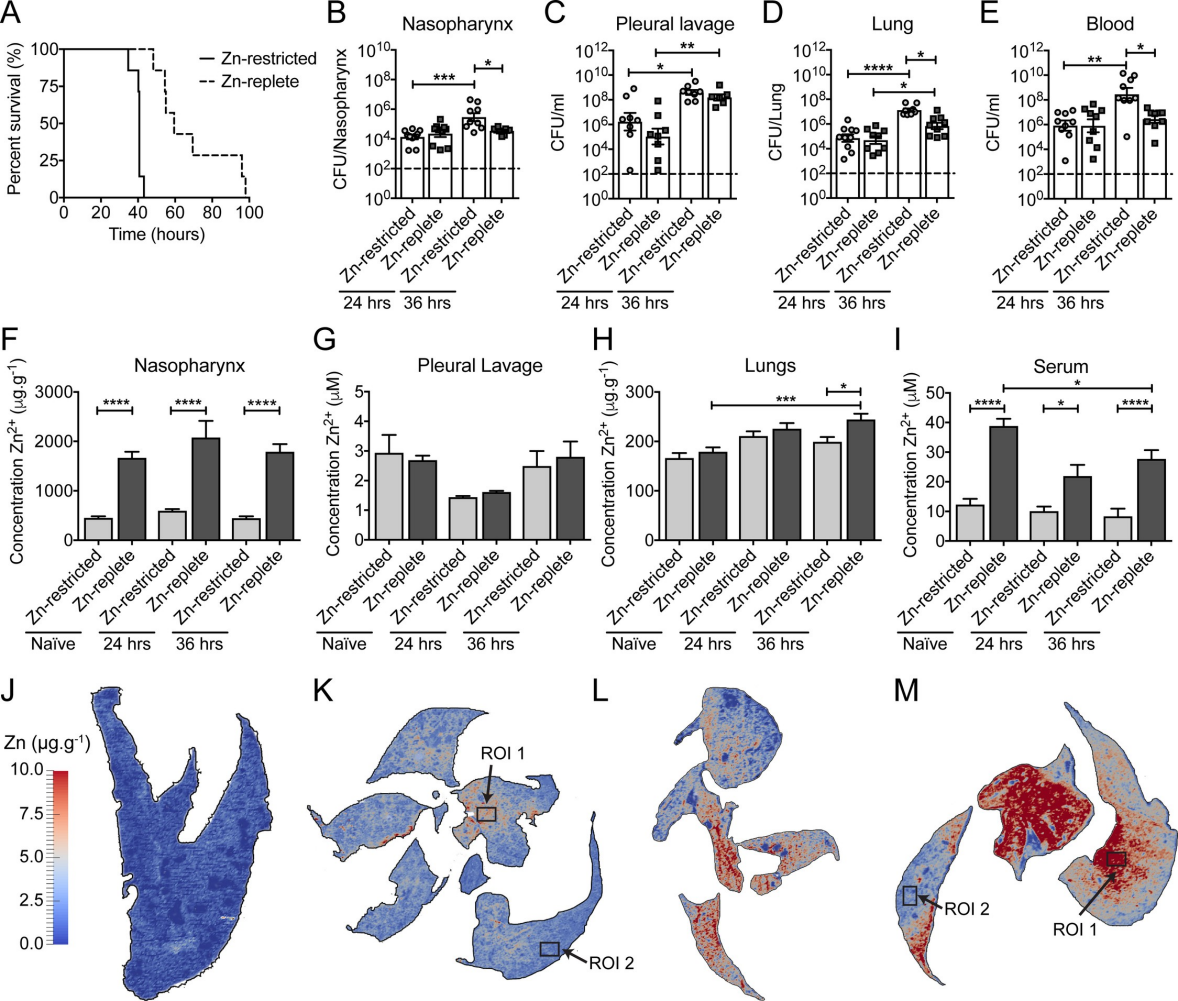
The effect of Zn restriction on animal survival, bacterial burden and tissue Zn abundance. (A) Survival of Zn-restricted or Zn-replete mice following intranasal challenge with 1 × 10^7^ CFU of *S*. *pneumoniae* strain D39. Data represent analyses of seven mice with statistical analyses performed using a Log-rank (Mantel-Cox) test. Enumeration of *S*. *pneumoniae* from host niches, nasopharynx (B), pleural lavage (C), lung (D), blood (E), following intranasal challenge with 1 × 10^7^ CFU of strain D39 (n ≥ 7). Colonisation was examined at 24 and 36 hrs post challenge in Zn-restricted or Zn-replete mice. Data represent the mean (± S.E.M.) of four independent experiments with statistical analyses performed by a one-way ANOVA. Determination of murine niche Zn abundance, nasopharynx (F), pleural lavage (G), lung (H), blood serum (I), prior to infection, or 24 hrs or 36 hrs post infection in both dietary groups (n ≥ 5) by ICP-MS. Data represent the mean (± S.E.M.) of four independent experiments with statistical analyses performed by a one-way ANOVA. Spatial distribution of Zn in the lungs of naïve Zn-restricted (J), or infected Zn-restricted (K) mice, and naïve Zn-replete (L) or infected Zn-replete (M) mice at 36 hrs post challenge. These data are representative elemental maps of murine tissue sections from analyses of at least three distinct murine samples (n ≥ 3) by laser-ablation-ICP-MS with quantitated regions of interest (ROI) highlighted. Mean (± S.E.M.) elemental data for the distinct tissue sections is presented in S2 Table. The scale bar represents a heat map (blue to red) for the intensity of Zn from 0 to 10.0 μg.g^-1^.

To investigate the role of host Zn in pneumococcal disease progression, we developed a murine model of Zn-deficiency. Dietary intervention achieved an approximate 70% reduction in serum Zn, compared to mice fed on the standard chow diet (12 μM to 40 μM; p < 0.0001, one-way ANOVA) (S1 Fig). Supplementation of drinking water with 250 ppm Zn was the minimum concentration required to restore serum Zn levels to that of mice fed on standard chow diet (S1 Fig). Analyses of sera of mice fed on the two diets, Zn-replete (250 ppm supplementation) and Zn-restricted (0 ppm supplementation), revealed that the impact on first row transition metal ion levels was restricted to Zn (S1 Table). We then investigated the impact of dietary Zn restriction on murine survival time after *S*. *pneumoniae* infection. Here, we performed an intranasal challenge with 1 × 10^7^ colony forming units (CFUs) of the *S*. *pneumoniae* serotype 2 strain D39. Mean murine survival time decreased by ~30 hrs for mice fed on the Zn-restricted diet, relative to mice fed on the Zn-replete diet (40.2 hrs vs. 70.9 hrs; p < 0.01, Student’s *t*-test) (Fig 1A). Together, these data show that dietary Zn influences host control of pneumococcal infection in our murine model.

### Dietary zinc and its impact on tissue zinc abundance and pneumococcal proliferation

Building on the above framework, we sought to ascertain how murine Zn levels influenced infection by *S*. *pneumoniae*. Bacterial burden was assessed at 24 and 36 hrs post-challenge in the clinically relevant niches of the nasopharynx, pleural cavity, lung and blood (Fig 1B–1E). At 24 hrs post-challenge, the Zn-restricted and Zn-replete mice showed no differences in bacterial burden in any of the niches (Fig 1B–1E). By 36 hrs, this had changed with the bacterial burden significantly increasing in the Zn-restricted mice. By contrast, the Zn-replete mice, whilst also demonstrating a general increase in bacterial burden relative to 24 hrs, showed a reduced extent of pneumococcal proliferation (Fig 1B–1E). This was most apparent in the nasopharynx and the blood, where the Zn-replete mice showed no increase in bacterial burden between 24 and 36 hrs, whereas in the Zn-restricted mice the pneumococcal burden increased by 1–2 orders of magnitude in the same niches (Fig 1B and 1E). Pneumococcal burden in murine lungs increased in both Zn-restricted and Zn-replete mice from 24 to 36 hrs (Fig 1D). However, the increase was significantly greater in Zn-restricted mice. The pleural cavity, assessed by lavage, was the only niche in which no differences were observed between the dietary groups at either time point (Fig 1C).

We then assessed niche Zn abundance and compared the two dietary groups (Fig 1F–1I). In Zn-restricted naïve mice, the nasopharynx and blood serum had significantly reduced Zn abundance, by comparison with the Zn-replete naïve mice (Fig 1F and 1I). In contrast, no significant differences in total Zn were observed for the pleural cavity or lungs. Mobilisation of Zn is a physiological component of systemic inflammation control and the acute phase response [44, 45]. Consequently, we investigated how Zn abundance changed over the course of pneumococcal infection. We observed that tissue Zn abundance did not significantly change in any niche of the Zn-restricted mice. Abundance of Zn was also unaltered in Zn-replete mice in the nasopharynx and pleural cavity during infection (Fig 1F and 1G). However, consistently higher Zn levels (4-fold) were observed in the nasopharynx of Zn-replete mice, compared to Zn-restricted mice (Fig 1F). Although, blood serum Zn levels decreased in Zn-replete mice upon infection, consistent with prior studies [46], they remained significantly elevated by comparison with Zn-restricted mice at 24 (2.2-fold) and 36 hrs (3.3-fold) post challenge (Fig 1I). The abundance of Zn also increased in the lungs of Zn-replete mice at 36 hrs post-challenge and was significantly greater than Zn-restricted mice (1.2-fold) (Fig 1H). We then sought to investigate the flux of Zn in the lungs during infection in greater detail.

**Fig 2:**
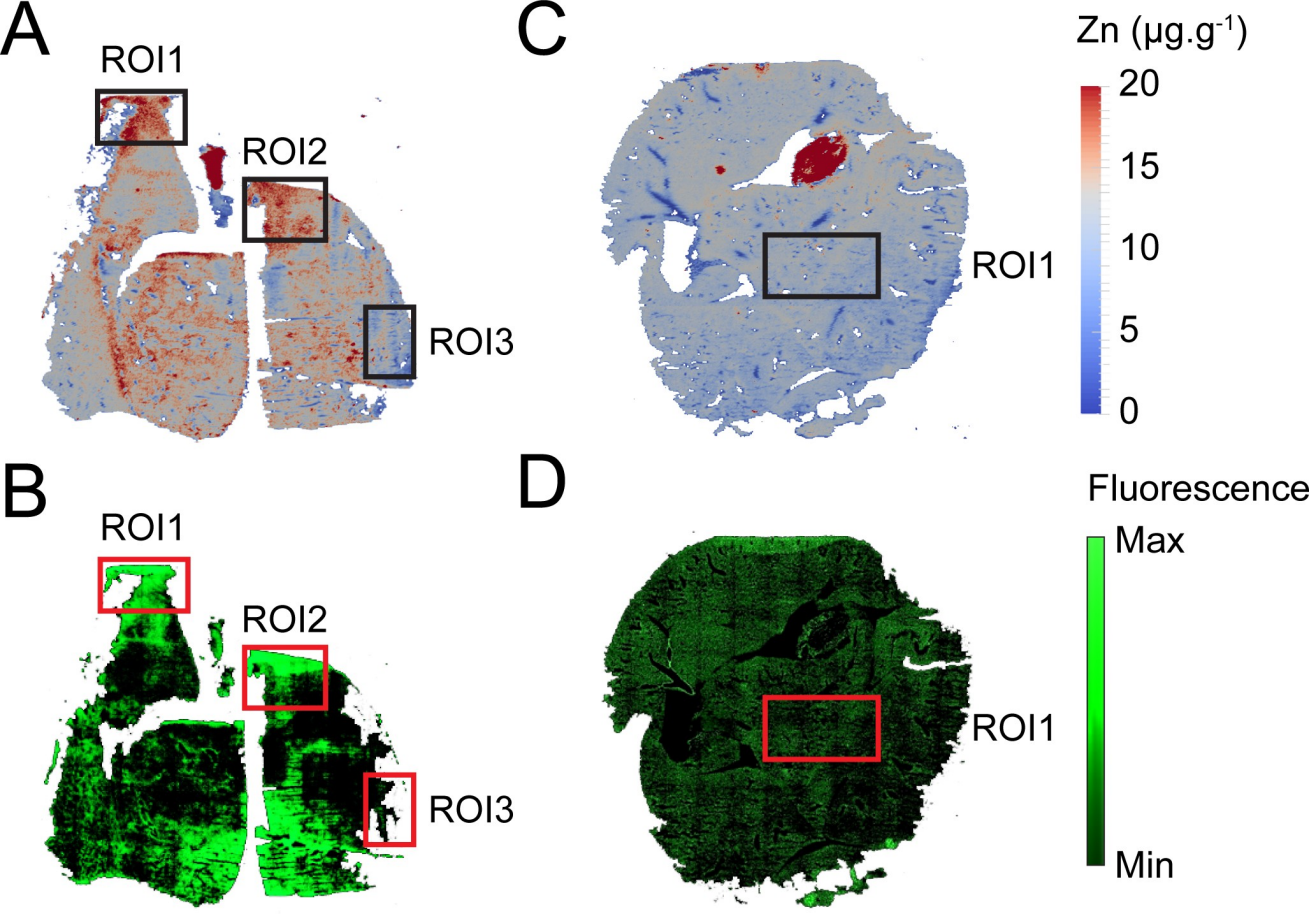
Co-localisation of *S*. *pneumoniae* and Zn in murine lung tissue. Spatial distribution of Zn in the lungs of infected Zn-replete (A) or naïve Zn-replete (C) mice at 36 hrs post challenge. These data are representative elemental maps of murine tissue sections from at least two distinct murine samples by laser-ablation-ICP-MS with quantitated regions of interest (ROI) highlighted. The scale bar represents a heat map (blue to red) for the intensity of Zn from 0 to 20.0 μg.g^-1^. Spatial distribution of *S*. *pneumoniae* pVA838-GFP fluorescence in the lungs of infected Zn-replete (B) or naïve Zn-replete (D) mice at 36 hrs post challenge. The data are representative murine tissue sections from at least two distinct murine samples analysed by fluorescence microscopy with regions of interest (ROI) highlighted. The scale bar represents a heat map (black to green) for the relative fluorescence intensity.

Elemental bio-imaging was used to map the spatial distribution of Zn in murine lung tissue (Fig 1J–1M). This revealed relatively even distribution of Zn throughout the lung tissue of naïve mice with mean Zn concentrations of 1.35 ± 0.53 and 7.86 ± 1.08 μg.g^-1^, for the restricted (Fig 1J) and replete (Fig 1L) tissues sections, respectively. This trend and the relative differences were consistent across multiple distinct analyses (S2 Table) and corresponded with dietary intervention and the whole-organ ICP-MS analyses. Upon infection, the mean Zn concentrations for the restricted and replete tissue sections were 2.56 ± 0.25 and 9.55 ± 1.21 μg.g^-1^, respectively, (Fig 1K and 1M and S2 Table), but distribution of Zn within the tissue sections was no longer uniform with the emergence Zn-enriched regions [region of interest (ROI)]. In Zn-restricted mice, the Zn enriched ROI (ROI 1 in Fig 1K) had a mean Zn concentration of 4.95 ± 0.08 μg.g^-1^. This reflects a ~2-fold increase in Zn abundance compared to the mean tissue section concentration and a ~3-fold increase by comparison to the naïve tissue. Conversely, in a region distant from Zn enrichment the concentration of Zn was reduced to 1.72 ± 0.04 μg.g^-1^ (ROI 2 in Fig 1K). This was significantly less than the average tissue concentration suggesting that, in addition to Zn influx to the lungs, Zn was redistributed within lung tissue. The emergence of Zn-enriched regions was also observed in the lungs of Zn-replete infected mice (Fig 1M). Here, the Zn concentration increased to 15.75 ± 0.04 μg.g^-1^ in an enriched region (ROI 1 in Fig 1M), ~1.6 fold greater than the mean tissue section concentration. Similar to Zn-restricted mice, Zn abundance also decreased in areas distant from the enriched regions with a concentration of 4.19 ± 0.09 μg.g^-1^ determined for a representative area (ROI 2 in Fig 1M). These data show that regions of selective enrichment and depletion within murine lungs were not uniform across the tissues and most likely varied due to bacterial infection. However, the spatial complexities occurred irrespective of diet, with the extent of change directly related to dietary Zn intake which in turn influenced Zn flux. The location of *S*. *pneumoniae* within the lung tissue of Zn-replete mice was then assessed using a strain expressing GFP [47]. Elemental bio-imaging revealed that regions with increased Zn abundance (>10 μg.g^-1^) corresponded with regions of increased GFP fluorescence (ROI 1 and ROI 2 in Fig 2A and 2B; ROI 1 and ROI 2 in S2A and S2B Fig). In contrast, regions with lower fluorescence corresponded to regions where Zn abundance was not significantly different from the average tissue concentration (ROI 3 in Fig 2A and 2B; ROI 3 in S2A and S2B Fig) or naïve Zn-replete lung tissue (ROI 1 in Fig 2C and 2D; ROI 1 in S2C and S2D Fig) (< 10 μg.g^-1^). Taken together, these data show that Zn co-localises with the invading pathogen in murine lungs.

Collectively, our analyses reveal an inverse correlation between host tissue Zn levels and the overall burden of pneumococci at 36 hrs post-challenge. Further, our data directly shows that Zn abundance increases in the lung upon infection and is predominantly localised in regions where *S*. *pneumoniae* is present and thus may contribute to control of pneumococcal proliferation.

### Murine Zn abundance directly impacts the pneumococcus

**Fig 3:**
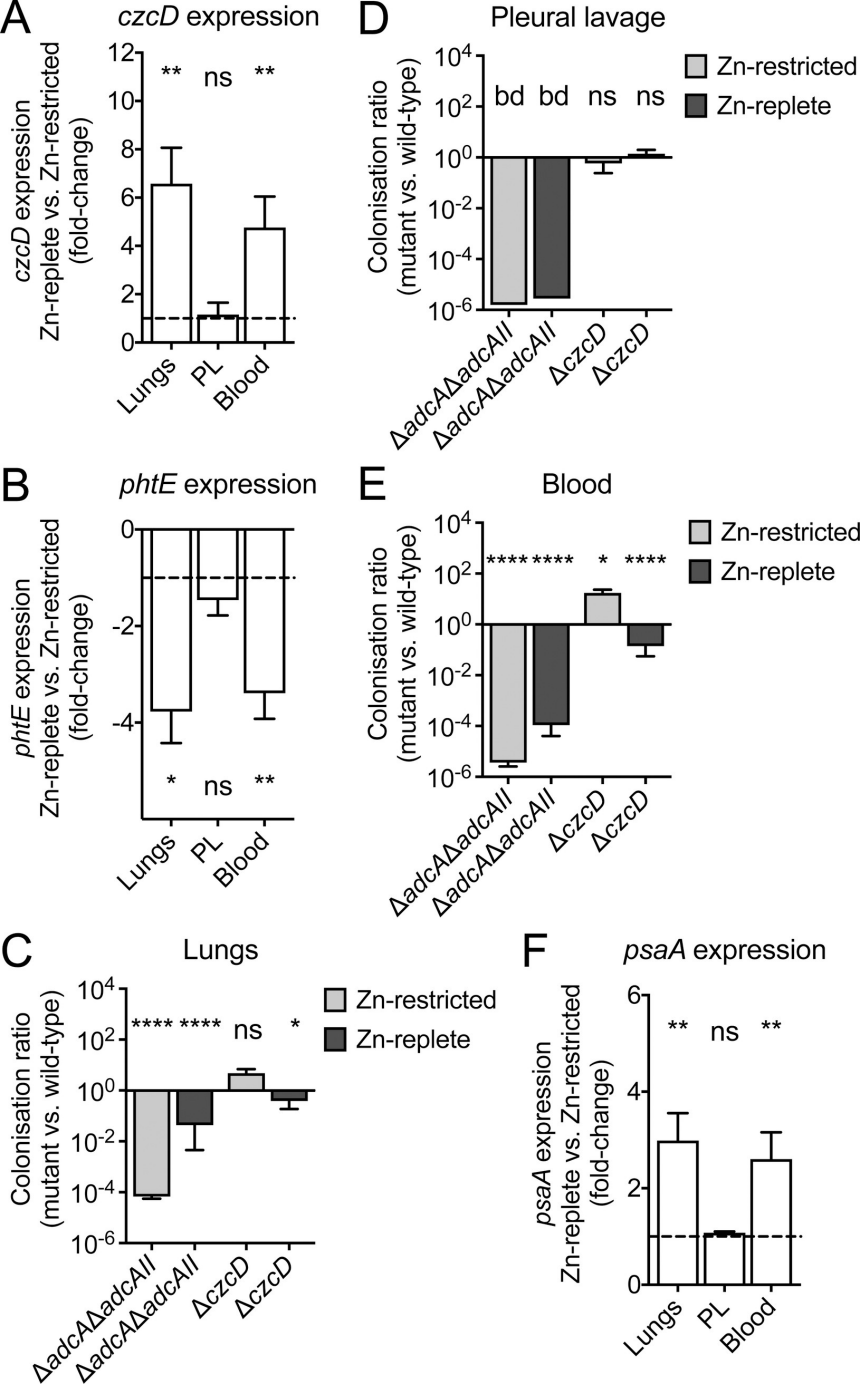
The effect of host dietary intervention on pneumococcal metal ion homeostasis. Pneumococcal expression levels of *czcD* (A), *phtE* (B) and *psaA* (F) were determined following isolation from the lungs, blood or pleural lavage (PL) of Zn-restricted and Zn-replete mice (n ≥ 6). Gene expression levels are the difference, in fold-change, of pneumococci from the Zn-replete mice as compared to pneumococci from the Zn-restricted mice. Data represent the mean (± S.E.M.) of two independent experiments with statistical analyses performed using Student’s *t*-test. The proliferation of pneumococcal Zn acquisition (Δ*adcA*Δ*adcAII*) and Zn efflux (Δ*czcD*) mutants was compared to that observed for wild-type pneumococci in the lungs (C), pleural lavage (D) and blood (E) at 36 hrs post challenge (n ≥ 5). Data represent the mean (± S.E.M.) of three independent experiments with statistical analyses performed by Student’s *t*-test.

We then sought to ascertain whether control of pneumococcal proliferation could be linked to host Zn abundance. This was addressed by profiling the transcription of two *S*. *pneumoniae* Zn homeostasis genes, *czcD* and *phtE*, during infection of mice in both dietary groups. CzcD is the primary Zn efflux transporter of *S*. *pneumoniae*, and its transcription is highly sensitive to increased intracellular Zn abundance resulting in up-regulation of expression [42, 48]. Conversely, PhtE is a component of the pneumococcal Zn-starvation response and is also highly responsive to intracellular Zn limitation, but is down-regulated [32]. Here, we isolated bacterial RNA from murine lungs, the pleural cavity and blood of Zn-replete and Zn-restricted mice at 36 hrs post-challenge. Transcriptional analyses revealed that *czcD* was significantly up-regulated in pneumococci in the lungs (6.6-fold) and blood (4.8-fold) of Zn-replete mice (p<0.01, Student’s t-test) (Fig 3A). Concordantly, *phtE* was significantly down-regulated in pneumococci colonising these same niches (lungs, 3.0-fold, p<0.05; blood, 3.1-fold, p<0.01; Student’s t-test) of Zn-replete mice (Fig 3B). By contrast, transcriptional analysis of pneumococci from the pleural cavity showed no differences in Zn-dependent gene transcription between the two dietary groups (Fig 3A and 3B). These findings are highly consistent with the niche tissue Zn analyses, which showed consistently higher Zn abundance in the Zn-replete mice for the lungs and blood and no differences for the pleural cavity (Fig 1). Further, these data reveal that pneumococci in the lungs and blood of Zn-replete mice are directly exposed to increased Zn levels in these niches.

To further examine how murine Zn abundance influenced bacterial infection we employed pneumococcal strains compromised, to differing extents, in Zn uptake (Δ*adcA*, Δ*adcAII*, Δ*adcA*Δ*adcAII*) and Zn efflux (Δ*czcD*) [32, 48]. Comparison of the bacterial loads (wild-type D39 versus mutant strains) in the lungs, pleural cavity and blood of Zn-replete and Zn-restricted mice were then performed. Deletion of the Zn-recruiting proteins in isolation, i.e. Δ*adcA* or Δ*adcAII*, had minimal effect on pneumococcal proliferation in either dietary group (S3A–S3C Fig). Mutation of both genes (Δ*adcA*Δ*adcAII*), which abrogates Zn uptake, significantly impaired invasive pneumococcal disease (Fig 3C–3E). The Δ*adcA*Δ*adcAII* mutant strain was not detected in the pleural cavity of mice from either dietary group (Fig 3D). This finding, when considered with the tissue Zn determination (Fig 1G) suggests that the pleural cavity is a niche of low Zn abundance irrespective of diet. In Zn-restricted mice, the mutant strain was also significantly impaired for infection of the lungs and blood by comparison to the wild-type strain (Fig 3C and 3E). By contrast, deletion of the pneumococcal Zn efflux transporter had strikingly different effects. In the pleural cavity, the Δ*czcD* strain colonised mice from both dietary groups to the same extent as the wild-type (Fig 3D). In the blood of Zn-restricted mice, the Δ*czcD* strain had 17-fold greater abundance than that of the wild-type (Fig 3E) suggesting that Zn is poorly available to *S*. *pneumoniae* in this niche. This inference is consistent with serum Zn analysis (Fig 1I) of Zn-restricted mice and the reduced fitness of the Δ*adcA*Δ*adcAII* strain in this niche. By contrast, the Δ*czcD* strain colonised the blood of Zn-replete mice to a lesser extent than the wild-type, consistent with increased niche Zn abundance in this dietary group (Fig 1I). In the lungs of Zn-restricted mice, the Δ*czcD* strain showed no significant difference in infection compared to the wild-type strain. However, in the lungs of Zn-replete mice the Δ*czcD* strain was 2.6-fold lower in abundance, compared to the wild-type strain. Decreased survival of the Δ*czcD* strain in the lungs of Zn-replete mice was consistent with the increased competitiveness of the Δ*adcA*Δ*adcAII* strain in this niche and greater Zn abundance shown by ICP-MS and elemental bio-imaging (Fig 1H and 1J–1M). Taken together, these data indicate that during infection the abundance of Zn in the lungs increases and this is directly encountered by invading pneumococci and influences infection kinetics.

Collectively, these data show that murine dietary Zn intake directly impacts the abundance of the metal ion encountered by invading pneumococci in various host niches and thereby, influences the Zn homeostatic pathways of the pathogen.

### Dietary Zn affects pneumococcal manganese acquisition

Manganese (Mn) acquisition in *S*. *pneumoniae* is facilitated by the ABC permease, PsaBCA [35, 49]. The function of this pathway can be disrupted by Zn, which has been shown to compete with Mn for binding to PsaA and block its import at physiologically relevant concentrations (S3D Fig) [31, 50–52]. Transcription of *psaBCA* is negatively regulated by Mn abundance and positively regulated by Zn abundance via the DtxR family regulator PsaR (S3E Fig) [53]. Here, we examined *psaA* transcription to ascertain whether murine niche Zn abundances were influencing pneumococcal Mn homeostasis (Fig 3F). We observed that *psaA* transcription was significantly up-regulated in pneumococci isolated from the lungs and blood of Zn-replete mice, by comparison with Zn-restricted mice (lungs 3.0-fold, p<0.01; blood 2.6-fold, p<0.01), but not in the pleural cavity. Analysis of the Mn abundance in these niches revealed that mice from the two dietary groups had similar Mn levels in their respective tissues (S3F–S3H Fig). Further, elemental bio-imaging of murine lungs from the two dietary groups also showed the same abundances and distribution of Mn both prior to and post-infection (S3I Fig and S2 Table). Therefore, up-regulation of *psaA* is not attributable to differences in host tissue Mn abundance.

Bioavailability of Mn can be altered during *in vivo* infection. One prominent mechanism is the S100-family protein calprotectin (S100A8/S100A9), which has a major role in nutritional immunity and has been identified in the bronchoalveolar lavage fluid of patients with pneumococcal pneumonia [54, 55]. Here, transcription of murine *S100A8*, which is co-transcribed with *S100A9*, was examined in the lungs and blood of mice from both dietary groups. In naïve mice, transcription of *S100A8* in the lungs and blood was not significantly different between dietary groups. Upon infection, expression of *S100A8* increased in both Zn-restricted and Zn-replete mice in the lungs and blood (S4A–S4D Fig). Although expression of calprotectin did not significantly differ in the lungs between the two dietary groups (S4A–S4C Fig), *S100A8* was up-regulated to a greater extent in the blood of Zn-restricted mice (2-fold; p<0.0001) (S4D Fig). This increase in calprotectin transcription may arise from the higher bacterial burden in this niche in Zn-restricted mice.

Collectively, these findings show that host Mn abundance is not affected by dietary Zn intake, nor does it appear to undergo significant changes in total abundance in the lungs or blood during infection. It therefore follows that host Zn abundance, influenced by diet, impacts pneumococcal Mn homeostasis during infection. In the context of Zn-replete mice, this results in a pattern of bacterial gene transcriptional responses consistent with Zn intoxication, i.e. up-regulation of *psaA* and *czcD* (S3E Fig), in the lungs of Zn-replete mice. Hence, these findings provide a plausible explanation for the lower bacterial burden observed in Zn-replete mice, by comparison to the Zn-restricted mice.

### The effect of dietary Zn on the immune response

**Fig 4:**
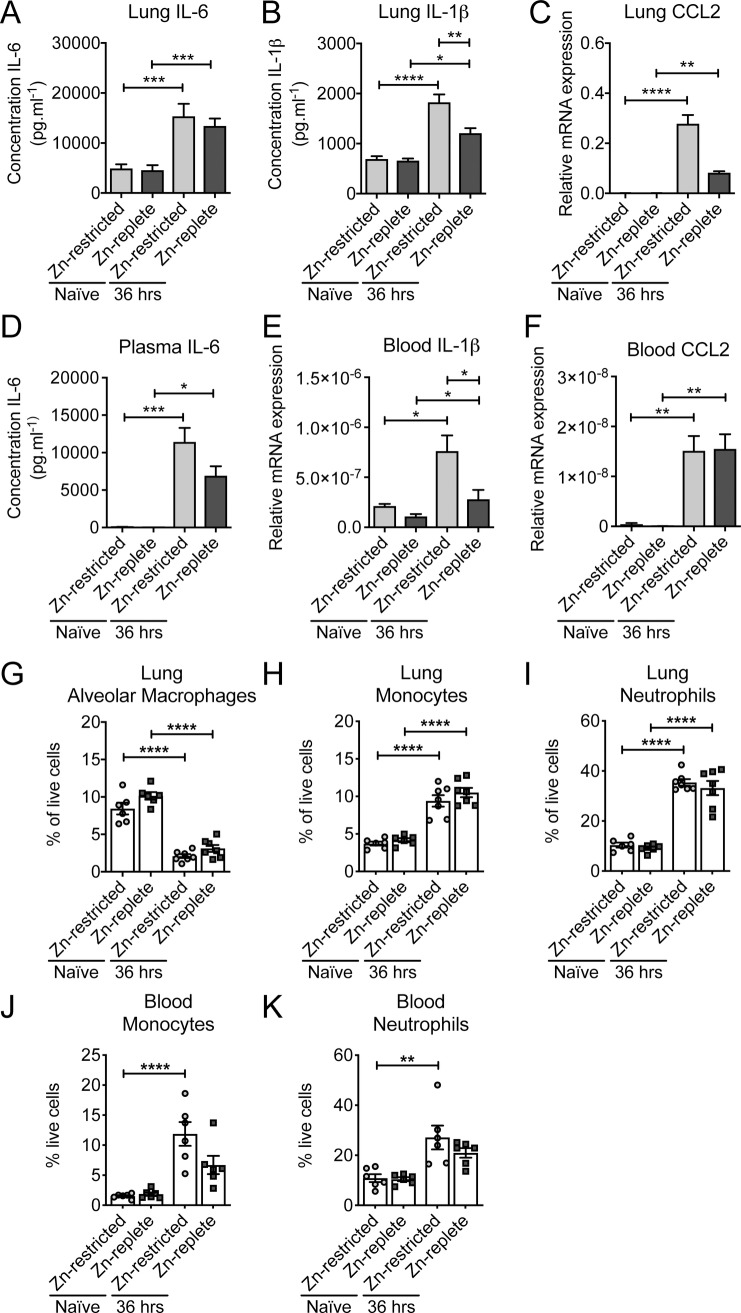
The effect of dietary Zn on the innate immune response. Quantitative ELISA analyses of IL-6, in the lungs (A) and blood (D), and IL-1β, in the lungs (B), in Zn-restricted and Zn-replete mice prior to or 36 hrs post infection. The data represent the mean (± S.E.M.) of at least four independent biological replicates with statistical analyses performed using a one-way ANOVA. Transcriptional profiling of the innate immune response for CCL2, in the lungs (C) and blood (F), and IL-1β, in blood (E), in Zn-restricted and Zn-replete mice (n ≥ 6) prior to or 36 hrs post infection by qRT-PCR. Data represent the mean (± S.E.M.) of two independent experiments with statistical analyses performed using a one-way ANOVA. Examination of cell abundances (percentage of live cells of total cells analysed) for alveolar macrophages, in the lungs (G), monocytes, in the lungs (H) and blood (J), neutrophils, in the lungs (I) or blood (K) of Zn-restricted and Zn-replete mice prior to (naïve) or 36 hrs post infection by flow-cytometry (n ≥ 5). Data represent the mean (± S.E.M.) of two independent experiments with statistical analyses performed using a one-way ANOVA.

Building on these findings, we sought to assess whether murine Zn levels influenced the immune response since Zn deficiency has previously been associated with dysregulated innate immune activation [56]. Here, we investigated the contribution of dietary Zn on the immune response to pneumococcal infection. Transcriptional profiling and cytokine analyses of the lungs and blood of infected Zn-replete mice revealed up-regulation of interleukins (IL) 1β and 6, and the chemokine CCL2, by comparison to naïve mice (Fig 4A–4F). This pattern of expression indicates an innate immune response associated with pneumococcal infection control and host defence [57–62]. Innate immune activation, and the extent thereof, is controlled by multiple negative feedback pathways [63]. NF-κβ, a crucial immune response modulator, has been shown to be negatively regulated by the IκB kinase (IKK) complex in a Zn-dependent manner. Prior studies of polymicrobial sepsis have shown that under conditions of Zn deficiency, the inability to supress NF-κβ activation leads to overproduction of the pro-inflammatory cytokines IL-1β and IL-6, resulting in septic shock [46]. Consequently, we examined whether Zn deficiency dysregulated this inflammatory axis in the context of pneumococcal infection by assessing transcription of *nfkb-1* (p105 subunit) and *nfkb-2* (p100 subunit). Comparison of the transcriptional profiles between the dietary groups revealed that expression of *nfkb-1* was not significantly influenced by diet or by infection in either the lungs or the blood (S5A and S5D Fig). By contrast, during infection transcription of *nfkb-2* was up-regulated to a similar extent in the blood, irrespective of diet, as well as in the lungs of Zn-restricted mice (S5B and S5E Fig). We further interrogated the role of NF-κβ by examining the phosphorylation status of the P65 (RelA) subunit of this protein, which is crucial for its activation. Quantitative immunoblotting revealed that there were no significant differences in the relative abundance of the phosphorylated form of NF-κβ between the dietary groups or upon infection (S5C and S5F Fig). Building on these analyses we compared the transcriptional and cytokine profiles between the two dietary groups post-infection. This revealed differences in the extent of up-regulated responses between the two dietary groups (Fig 4A–4F). Notably, IL-1β was elevated in the lungs and blood and CCL2 in the lungs of Zn-restricted mice, by comparison to Zn-replete mice (Fig 4B, 4C, and 4E). Taken together, these data show that Zn-restriction is associated with a greater inflammatory cytokine response, although without dysregulation of NF-κβ signalling, to pneumococcal infection, albeit in the presence of a greater bacterial load.

Collectively, the above data indicate that the failure of Zn-restricted mice to control *S*. *pneumoniae* infection was not due to a failure in activation of the innate immune response. Consequently, we sought to examine whether, upon dietary restriction of Zn, innate immune cell recruitment was affected and the impact, if any, on *S*. *pneumoniae* clearance. We first examined the abundance of alveolar macrophages, monocytes and neutrophils in the Zn-restricted and -replete mice both prior to and 36 hrs post-infection. Residing alveolar macrophages play a primary role during the very early stages of infection by identifying and opsonizing the invading pathogen and activating the required immune response that will lead to an influx of phagocytic cells [64]. Our data shows that their abundance is not affected by dietary intervention (Fig 4G). Upon infection, a relative decrease in the abundance of alveolar macrophages was observed in both the Zn-restricted and Zn-replete mice, possibly due to the infiltration of other major phagocytic cells such as monocytes and neutrophils. Despite this, there was no significant difference between the two dietary groups. Monocyte and neutrophil abundance increased by more than 2-fold post-infection in murine lungs with no apparent differences between the dietary groups (Fig 4H and 4I). In the blood, monocytes and neutrophils were also observed to increase, ~4-7-fold and ~2-fold, respectively, in both Zn-restricted and Zn-replete mice in response to pneumococcal infection. Nonetheless, there were no significant differences between the dietary groups (Fig 4J and 4K) indicating that murine Zn status does not affect the relative abundances of phagocytic cells prior to or post-infection.

Collectively, these data indicate that although dietary Zn deficiency does influence, to a minor extent, murine innate immune activation, when considered in the context of phagocytic cell recruitment the results do not suggest a generalised failure or dysregulation of the innate immune response to pneumococcal infection. Therefore, it follows that the increased pneumococcal burden arises from an impaired ability to prosecute clearance of the pathogen.

### The contribution of Zn to immune cell-mediated killing of pneumococci

**Fig 5:**
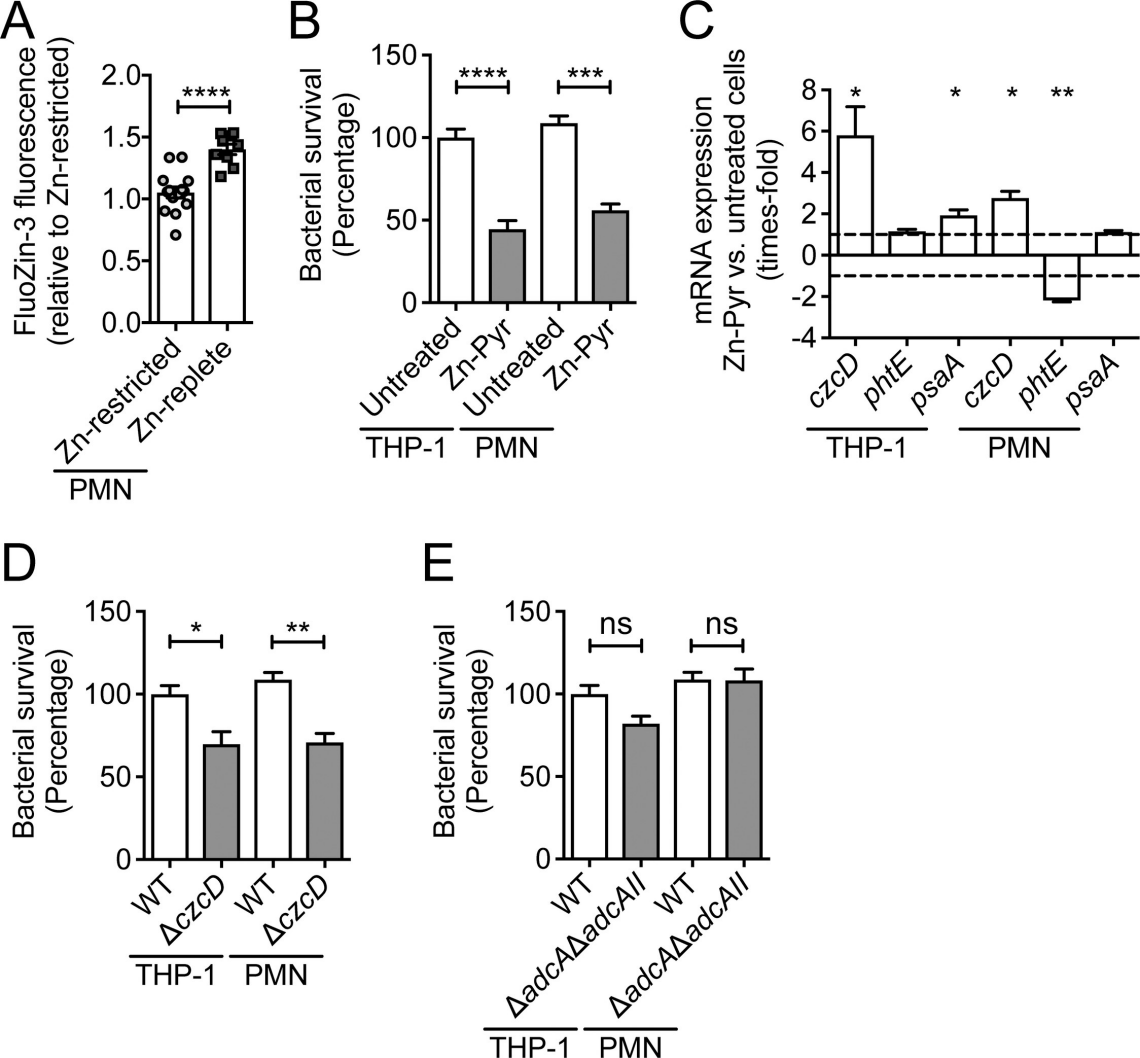
The role of Zn in phagocyte-mediated killing of *S*. *pneumoniae*. (A) Intracellular Zn in PMNs from Zn-restricted or Zn-replete mice examined using FluoZin-3 AM and flow-cytometry (n ≥ 5). Relative fluorescence was corrected to levels observed in the phagocytic cells in Zn-restricted mice. Data represent the mean (± S.E.M.) of two independent experiments with statistical analyses performed by Student’s *t*-test. (B) Survival of wild-type *S*. *pneumoniae* D39 in THP-1 monocyte-derived macrophages (n = 5) and human PMNs (n = 3) in the presence of Zn [50 μM Zn-pyrithione (Zn-pyr)] supplementation, by comparison with unsupplemented phagocytic cells. Data represent the mean (± S.E.M.) of three independent experiments with statistical analyses performed by Student’s *t*-test. (C) Expression levels of *czcD*, *phtE* and *psaA* in *S*. *pneumoniae* in THP-1 monocyte-derived macrophages (n = 5) and human PMNs (n = 3) with or without 50 μM Zn treatment. Expression levels represent the fold-change difference between pneumococci in the Zn-supplemented phagocytic cells compared to untreated cells. Data represent the mean (± S.E.M.) of three independent experiments with statistical analyses performed by Student’s *t*-test. Survival of *S*. *pneumoniae* mutant strains defective in Zn efflux (Δ*czcD*) (D) or Zn uptake (Δ*adcA*Δ*adcAII*) (E) in THP-1 monocyte-derived macrophages (n = 5) and human PMNs (n = 3), compared to the wild-type strain. Data represent the mean (± S.E.M.) of three independent experiments with statistical analyses performed by Student’s *t*-test.

Zinc has been shown to contribute to immune-cell mediated killing of various bacterial pathogens [45]. Although dietary Zn intervention did not impair immune-cell recruitment, we ascertained the effect on the intracellular Zn-status of the major phagocytic cells. Blood leukocytes were incubated with the cell permeable Zn-sensing probe, FluoZin-3 AM, followed by flow-cytometric quantitation of fluorescence in PMNs. These analyses indicated that Zn levels were significantly higher within polymorphonuclear (PMN) cells of Zn-replete mice compared to Zn-deficient mice (p<0.0001) (Fig 5A). To examine how intracellular Zn influenced pneumococcal survival, we pre-treated primary human polymorphonuclear cells and THP-1 derived macrophages with Zn (50 μM Zn-pyrithione) and subsequently examined pneumococcal loading compared to untreated cells. Here, we observed that Zn supplementation significantly reduced bacterial survival in PMNs (p<0.001) and THP-1 cells (p<0.0001) by comparison with untreated cells (Fig 5B). We then sought to ascertain whether the phagocytosed bacteria were being directly exposed to intracellular Zn. This was addressed by transcriptional profiling of the pneumococcal genes *czcD*, *phtE* and *psaA*, which are indicative of Zn intoxication, Zn limitation and Mn starvation, respectively (Fig 5C). In Zn-supplemented THP-1 macrophages, *czcD* and *psaA* transcription were significantly up-regulated by comparison to untreated controls (both p<0.05). In human PMNs, Zn supplementation resulted in up-regulation of *czcD* and down-regulation of *phtE* (p<0.05 and p<0.01, respectively). These data show that Zn-supplemented phagocytic cells expose the captured pneumococci to higher levels of intracellular Zn than untreated cells. We then investigated the impact of the increased Zn exposure on survival of phagocytosed *S*. *pneumoniae*. Here, we compared mutant strains deficient in either Zn-uptake (*ΔadcAΔadcAII*) or Zn-efflux (*ΔczcD*) in PMNs and THP-1 macrophages. We observed that the *ΔczcD* strain had reduced survival in both THP-1 macrophages and PMNs compared to the wild-type (p<0.05 and p<0.01, respectively; Fig 5D). By contrast, the *ΔadcAΔadcAII* strain was unaffected (Fig 5E). Collectively, our data show that restriction of dietary Zn reduces phagocytic cell Zn abundance, which in turn compromises the efficacy of *S*. *pneumoniae* killing due to the inability to employ Zn as a direct antimicrobial agent. Hence, these findings provide a mechanistic basis for the link between dietary Zn deficiency and increased susceptibility to pneumococcal infection.

## Discussion

Zinc deficiency compromises immune defence and leads to increased susceptibility to bacterial infections [65]. Here we have built upon previous observations, wherein murine Zn deficiency has been shown to promote invasive pneumococcal lung disease [25, 26], to elucidate how host Zn contributes to antimicrobial control of bacterial infection. We report that dietary Zn restriction significantly impacts Zn homeostasis in most, but not all, of the niches colonised by *S*. *pneumoniae*. These changes were specific to Zn, with other transition metal ion abundances unaltered by dietary intervention. Niches in which tissue Zn levels were reduced showed increased bacterial burden with the overall impact of dietary zinc restriction reducing murine survival time in response to *S*. *pneumoniae* challenge. Concordantly, in niches where the dietary intervention did not impact Zn status, such as the pleural cavity, pneumococcal burden remained unchanged between dietary groups. Collectively, our findings show that host Zn contributes to control of bacterial burden, although its flux is spatially and temporally complex.

Investigation of these changes revealed that they were niche-specific and influenced the progression of disease. In murine blood there was no level of reduction in serum Zn in either dietary group that prevented infection of this niche. Therefore, the pneumococcal Zn-specific uptake pathway is necessary and sufficient to acquire Zn from the blood, irrespective of host nutritional status and/or metal-withholding mechanism(s). Mobilisation of Zn from the blood into other tissues has previously been associated with host control of infection [5]. In this work, the only niche that had a concomitant increase in Zn abundance was the lungs. Elemental bio-imaging of this organ revealed infection-associated spatial flux of metal ions with the emergence of discrete regions enriched for Zn, but unaltered with respect to Mn. These analyses suggest that reduction in serum Zn levels, due to dietary intervention, impairs the mobilisation of Zn into the lungs during infection, thereby compromising host resistance. These findings are starkly different to observations from elemental bio-imaging of tissue abscesses associated with *Staphylococcus aureus* infection, wherein calprotectin-mediated withdrawal of Mn ions has been shown [66]. The formation of tissue micro-environments enriched for Zn, but unaltered with respect to other ions, suggests that regions of Zn intoxication occur within the lung during pneumococcal infection. Co-localisation, transcriptional and mutant infection studies show that the pathogen is directly exposed to these Zn-enriched regions, with pneumococcal metal ion homeostasis impacted in a Zn-dependent manner. Collectively, these findings indicate that Zn contributes to antimicrobial control within the lung during pneumococcal infection. The influx of antimicrobial Zn also provides a plausible explanation for the prior observation that murine control of *S*. *pneumoniae* lung infection was enhanced in calprotectin deficient mice [55]. The absence of calprotectin would permit Zn to act without sequestration by this host metal-withholding protein. However, this inference remains speculative as loss of *S100A9* has also been shown, albeit in a distinct model, to diminish control of pneumococcal infection due to impaired recruitment of neutrophils to the lung and reduced expression of pro-inflammatory cytokines [67]. Nonetheless, the broader significance of this work is that it highlights not only how diet influences infection dynamics and elemental abundances in niche-specific ways, but also reveals that whole-organ analyses can mask dramatic and complex changes within tissue microenvironments that are relevant to host control of infection.

Our findings suggest that mobilisation of Zn from serum to the lungs may be facilitated, at least in part, by phagocytic cells as a component of the innate immune response to infection. The Zn status of PMNs was significantly lower in Zn-restricted mice compared to the Zn-replete group. Despite the difference in cellular Zn status, the relative abundances of innate immune cells did not differ between the dietary groups prior to, or 36 hrs post infection. This was consistent with the transcriptional profiling of murine responses to pneumococcal infection, which revealed alterations in a subset of inflammatory-response associated genes between the dietary groups. This observation is notable as it is in stark contrast to prior studies of bacterial sepsis, wherein host Zn has been shown to play a key regulatory role in modulating NF-κβ-mediated inflammatory responses [46]. Here, restriction of dietary Zn intake resulted in only an increase in the activation of IL-1β and IL-6, but without infection resulting in a generalised dysregulation of inflammatory responses. However, as the pneumococcal burden was 1–2 orders of magnitude higher in Zn-restricted mice, the heightened immune activation is likely influenced by both host Zn status and bacterial burden. Nevertheless, the distinct differences in immune response between *S*. *pneumoniae*, a respiratory pathogen, and polymicrobial sepsis, induced by caecal ligation and puncture, reflect the differing modes of infection and host response. These data also show that control of pneumococcal infection was compromised in Zn-restricted mice, despite the lack of dietary impact on immune activation or phagocytic cell abundance. Impaired resistance to pneumococcal infection can be attributed to the contribution of Zn to phagocytic cell killing of bacterial pathogens [43, 68, 69]. Although the number and type of phagocytic cells present at the sites of pneumococcal infection did not differ between mice fed on the two diets, the Zn status of these cells was reduced in Zn-restricted mice. Thus, this study establishes a direct connection between host dietary intake, Zn status of phagocytic cells and their efficacy of bacterial killing. Investigation of how phagocyte Zn status influenced the killing of wild-type and mutant pneumococci revealed that bacterial Zn and Mn homeostasis was directly impacted, consistent with a Zn intoxication mechanism. Hence, this work links dietary Zn intake to the mechanism of bacterial control at a cellular level. Although Zn is not the sole determinant of bacterial control, acting in concert with other factors [68, 69], this work reveals how its deficiency compromises host control of infection and its antimicrobial mechanism against *S*. *pneumoniae*.

Collectively, this study elucidates how dietary Zn intake influences susceptibility to pneumococcal infection. Our work shows that this arises from Zn-poor diets providing inadequate Zn for mobilisation into tissues, particularly during infection, and the associated failure of phagocytic cells to efficaciously prosecute killing of pneumococci. Hence, this work highlights the importance of ensuring dietary Zn sufficiency as a critical component of population-wide resistance against the global burden of pneumococcal disease in conjunction with vaccination and other antimicrobial strategies.

## Materials and methods

### Ethics statement

All murine experiments were approved by the University of Adelaide Animal Ethics Committee (Animal Welfare Assurance number A5491-01; project approval number S-2013-053) and were performed in strict adherence to guidelines dictated by the Australian Code of Practice for the Care and Use of Animals for Scientific Purposes. Mice were anaesthetized by intraperitoneal injection of pentobarbital sodium (Nembutal; Rhone-Merieux) at a dose of 66 μg.g body weight^-1^. Mice were euthanized by CO_2_ asphyxiation. All experiments involving humans were performed in accordance with guidelines for low risk/negligible risk projects and approved by the Faculty of Science sub-committee of the University of Queensland Human Research Ethics Committee. Human neutrophils were obtained from healthy adult volunteers who provided informed written consent.

### Murine experiments

Two-week old outbred female CD1 (Swiss) mice were fed a low Zn diet (Specialty Feeds, WA, Australia) for two weeks, with the acidified drinking water supplemented with either 0 ppm or 250 ppm ZnSO_4_. For challenge with *S*. *pneumoniae* strain D39, *S*. *pneumoniae* D39 pVA838-GFP, or its mutant derivatives, treatment cohorts, comprised of at least 5 mice, were anaesthetized by intraperitoneal injection of pentobarbital sodium at a dose of 66 μg.g body weight^-1^, followed by intranasal administration of 30 μL bacterial suspension containing approximately 1 × 10^7^ CFU. The challenge dose was confirmed retrospectively by serial dilution and plating on blood agar. For survival experiments, mice were monitored regularly for signs of illness and euthanized once moribund. For determination of bacterial loads, at 24 or 36 hrs post-challenge, mice were euthanized by CO_2_ asphyxiation. Blood was collected by syringe from the posterior vena cava. The pleural cavity was lavaged through the diaphragm and the lungs through the trachea, both with 1 mL sterile PBS. Pulmonary vasculature was perfused by infusion of sterile PBS through the heart and lungs were subsequently excised. Lastly, the nasopharynx/upper palate was excised (nasopharyngeal tissue). Tissues were homogenized (Precellys Homogeniser) and all samples were serially diluted and plated on blood agar for bacterial counts. For metal ion determination by inductively coupled plasma mass spectrometry (ICP-MS), the tissues were homogenized, and all samples were boiled in HNO_3_ at the highest percentage achievable for the relevant niche, i.e. blood, and lung and nasopharyngeal tissue in 35% HNO_3_, and PL in 17.5% HNO_3_. Following removal of debris by centrifugation, samples were diluted to a final concentration of 3.5% HNO_3_. Metal ion detection by ICP-MS was perform on an Agilent 7500cx inductively coupled plasma-mass spectrometer (Adelaide Microscopy).

### Bacterial analyses

For RNA extraction and qRT-PCR analyses of pneumococci in infected mice, blood, PL and homogenized lung tissues were diluted in Bacterial RNA Protect (Qiagen) and centrifuged at 400 × *g* for 5 min at 4°C to remove the majority of eukaryotic cells and tissue debris. Bacterial cells were then collected by centrifugation at 3000 × *g* for 15 min at 4°C. For analysis of transcription levels of phagocytosed pneumococci, THP-1 cells or isolated human neutrophils were coincubated with *S*. *pneumoniae* strain D39 for 90 min, then washed and treated with 10 μg.mL^-1^ penicillin and 200 μg.mL^-1^ gentamycin for 30 min. The bacterial RNA was stabilized, and the eukaryotic cells lysed by incubation with Bacterial RNA Protect (Qiagen) for 5 min at RT. For all sample types, the bacterial RNA was extracted using hot-phenol followed by further purification over a RNeasy spin column (Qiagen). The total RNA samples were treated with DNase I (Roche) and qRT-PCR was carried out using a SuperScript III One-Step RT-PCR kit (Thermo Fisher Scientific) on a LC480 Real-Time Cycler (Roche). Transcription levels of genes analysed, were normalized to those obtained for 16S rRNA. Primer sequences are available in S3 Table. Results represent the mean (± S.E.M.) of two independent experiments each comprised of treatment cohorts with at least 6 mice, with statistical analyses performed using a one-way ANOVA (Graphpad Prism V7.0d).

Bacterial growth for metal content analyses was performed in cation-defined media (CDM), which corresponded to the C + Y media without transition metal-ion supplementation [51]. The base ion content of the CDM was ascertained by ICP-MS, as described previously [51]. CDM was then supplemented with MnSO_4_ and ZnSO_4_ at the concentrations specified. Cells were grown in 50 mL of culture to mid-log phase (absorbance at 600 nm = 0.3), harvested and prepared for analysis by ICP-MS, as described previously [51].

### FACS to examine the abundance of innate immune cells in the blood and lungs

Peripheral blood was collected into heparinised tubes from mice by cardiac puncture. Red blood cells were lysed for 5 min at 37°C using mouse red cell lysis buffer, and cells were then extensively washed and then resuspended at 4 × 10^6^.mL^-1^ in PBS with 1% BSA. Following transcardial perfusion, lungs were excised followed by treatment with 125 μg.mL^-1^ liberase and 100 μg.mL^-1^ DNase I in HBSS for 30 min at 37°C. The homogenized mix was passed through a 40 μm cell strainer and washed through using RPMI. Following washing, red blood cells were lysed at 37°C for 10 min using red blood cell lysis buffer. Cells were washed and resuspend in PBS with 1% BSA. The viability of lung and blood cells was examined using a LIVE/DEAD stain (Molecular Probes) at RT for 20 min. Following washing, Fc receptors were blocked by incubation with 400 μg.mL^-1^ murine gamma globulin (Rockland) for 15 min on ice before addition of fluorochrome conjugated monoclonal antibodies (CD45 (APC Cy7), CD11b (PE-Cy7), Gr1 (PE), MHCII (AF647), Siglec-F (BV421) [BD Biosciences] and F4/80 (AF488) [Invitrogen] or CD45 (APC), CD11b (PE-Cy7), Gr1 (FITC) and NK1.1 (PerCP Cy5.5) [BD Biosciences]) using the gating strategies shown in S6 and S7 Figs. Cells were washed after incubation for 30 min at 4°C. Cells were acquired on a BD LSR II and analysed using Flowjo software. Blood inflammatory monocytes: CD45^+^CD11b^+^F4/80^+^Gr1^+^, Blood neutrophils: CD45^+^CD11b^+^F4/80^-^Gr1^+^MHCII^+^ upon inflammation, Alveolar macrophages: CD45^+^F480^+^CD11b^low/neg^CD11c^+^Siglec F^+^, Lung inflammatory monocytes: CD45^+^CD11b^+^F480^+^Gr1^+^ and Lung neutrophils: CD45^+^CD11b^+^F4/80^-^Gr1^+^MHCII^+^ upon inflammation.

### FACS to determine the Zn status of phagocytic cells

Peripheral blood was collected into heparinized tubes from mice by cardiac puncture. Red blood cells were lysed for 5 min at 37°C using mouse red cell lysis buffer, and cells were then extensively washed and then resuspended at 4 × 10^6^ cells.mL^-1^ in PBS with 1% BSA. To determine the Zn status of PMNs, cells were labelled with Fluozin-3 AM (Molecular Probes) for 30 min at 37°C and washed in PBS with 1% BSA. Following this, Fc receptors were blocked by incubation with 400 μg.mL^-1^ murine gamma globulin (Rockland) for 15 min on ice before addition of fluorochrome conjugated monoclonal antibodies (CD115-APC, CD11b-BV421, Ly-6C-BV510, CD45-PE/Cy7 [Biolegend] and Gr1 (PE) [BD Biosciences]) and fixable live/dead reagent (Molecular Probes). Cells were stained for 30 min on ice in the dark, washed twice in cold PBS and then fixed in PBS with 1% PFA before analysis. Cells were acquired on a BD FACSAria and analysed using Flowjo software using the gating strategy shown in S8 Fig. PMNs were identified as live CD45^+^CD11b^+^Ly6C^int^Gr-1^hi^. The fluorescence intensity of these cells with respect to Fluozin-3 staining was quantified.

### Transcriptional analyses of murine samples

Prior to extraction of RNA using a RNeasy spin column (Qiagen), blood from the posterior vena cava was treated with red blood cell lysis buffer and lung tissues were treated with RNA*later* solution (Ambion). All samples were DNase I treated (Qiagen) on the column during purification. For examination of SA100A8 transcripts, qRT-PCR analyses were performed using a SuperScript III One-Step RT-PCR kit (Thermo Fisher Scientific) on a QuantStudio 7 Real-Time Cycler (Thermo Fisher Scientific). Transcription levels of genes analysed were normalized to those obtained for *ACTB*. Data represent the mean (± S.E.M.) of two independent experiments with treatment cohorts comprised of at least six mice. The examination of mouse immune markers by qPCR was conducted using the TaqMan Array Mouse Immune Panel (Thermo Fisher Scientific) on a QuantStudio 7 Real-Time Cycler (Thermo Fisher). The TaqMan Arrays analyses were performed in triplicate, with each sample representing RNA pools from at least three mice. The results were analysed using the ThermoFisher Cloud Software. For the TaqMan Arrays analyses, data represent the mean (± S.E.M.), with statistical analyses performed using a one-way ANOVA.

### ELISAs

The IL-6 and IL-1β levels were determined in mouse plasma and homogenized lung tissue supernatant, all according the manufacturer’s recommendations (ELISAKIT.COM). The results were analysed on a PHERAstar plate reader (BMG Labtech). Data represent the mean of at least four independent biological samples (mean ± S.E.M.), with statistical analyses performed using a one-way ANOVA (Graphpad Prism V7.0d).

### Phospho-P65 and P65 immunoblot analyses

Blood samples for analyses of Phospho-P65 and P65 immunoblots were collected from the posterior vena cava and treated with red blood cell lysis buffer, while lung tissues were homogenized using a tissue homogeniser (Precellys). Both blood and lung samples were further treated by sonication in a Bioruptor (Diagenode), following the manufacturer’s recommendations. Protein concentrations were determined (DC Bio-Rad protein assay; Bio-Rad), and 20 μg total protein was loaded into each lane of a 4–12% NuPage Bis-Tris gel (Invitrogen). After electrophoretic separation by SDS-PAGE, the proteins were transferred to a nitrocellulose membrane using an iBlot system (Thermo Fisher Scientific). Blots were incubated with rabbit anti-mouse phosphor-P65 or P65 (Cell Signalling Technology). This was followed by incubation with goat anti-rabbit IRDye 800 and analysis using an Odyssey infrared imaging system (LI-COR). Band intensities were measured using the manufacturer’s application software. Results are the mean ratios between phosphor-P65 and total P65 from four independent experiments (mean ± S.E.M.), with statistical analyses performed using a one-way ANOVA (Graphpad Prism V7.0d).

### Macrophage killing assays

THP-1 cells (ATCC TIB-202) were grown under atmospheric control (95% air and 5% CO_2_) at 37°C in complete RPMI medium (RPMI with phenol red [Gibco], supplemented with 10% fetal bovine serum, 10 mM HEPES, 30 μg.mL^-1^ penicillin and 50 μg.mL^-1^ streptomycin). Cell culture flasks (25 cm^2^; BD Falcon) were seeded with 3.5 × 10^6^ THP-1 cells and differentiated by adding 100 ng.mL^-1^ phorbol 12-myristate 13-acetate (PMA; Sigma-Aldrich) and incubated for three days. Attached differentiated THP-1 cells (macrophages) were washed in complete RPMI and incubated with complete RPMI without added PMA to allow resting for a minimum of 2 days. Two hours before challenge with *S*. *pneumoniae*, a subset of macrophages was treated with 50 μM zinc-pyrithione. Following thorough washing, THP-1 cells were detached using 1 mL StemPro Accutase (Thermo Fisher Scientific), washed in Hank’s Balanced Salt Solution (HBSS; Thermo Fisher Scientific) and diluted to 1.1 × 10^5^ cells.mL^-1^ in HBSS. Wild-type *S*. *pneumoniae* D39 and the *czcD* and *adcA/AII* mutants were grown overnight on blood agar plates at 37°C with 95% air and 5% CO_2_ and subsequently inoculated into C+Y media to an optical density at 600 nm (OD_600_) of 0.05. The cultures were grown until the OD_600_ reached 0.3, after which the cells were washed, resuspended in HBSS, and colony forming unit (CFU) counts determined by plating on blood agar. The macrophages and *S*. *pneumoniae* cells were co-incubated at a ratio of 1:10 for 90 min. The macrophages were then washed, and extracellular bacteria were killed by incubation with 200 μg.mL^-1^ gentamycin and 10 μg.mL^-1^ penicillin for 30 min. The macrophages were washed in HBSS without antibiotic and incubated for a further 60 min prior to analysis of intracellular bacteria by lysing the macrophages with 0.0625% Triton-X-100. The lysate was then plated onto blood agar. The CFUs were enumerated and corrected for input. Data represent the mean (± S.E.M.) of three experiments with five independent biological replicates. The statistical differences between pneumococcal survival in zinc-pyrithione treated and untreated THP-1 cells and between survival of wild-type and *czcD* or *adcA/AII* mutant pneumococci were examined using an unpaired Student’s *t*-test (Graphpad Prism V7.0d).

### Neutrophil killing assays

Human neutrophils were purified from venous blood from healthy candidates using PolyMorphPrep (Axis-Shield, Norway) as previously described [70]. The neutrophil killing assay was performed using mid-logarithmic phase (OD600 = 0.25–0.3) *S*. *pneumoniae* diluted in RPMI + 10% heat-inactivated human serum that was incubated with the purified neutrophils at a multiplicity of infection of 10:1 (*S*. *pneumoniae*:PMN) for 60 min. The PMNs were then lysed by diluting them in distilled water and *S*. *pneumoniae* plated to enumerate colonies. PMNs were preloaded with Zn by incubation with 50 μM zinc-pyrithione (Merck) for 30 min at room temperature, followed by washing with 1× PBS prior to use in the killing assays. Data represent the mean (± S.E.M.) of three experiments with three independent biological replicates. The statistical differences between pneumococcal survival in zinc-pyrithione treated and untreated PMNs and between survival of wild-type and *czcD* or *adcA/AII* mutant pneumococci were examined using an unpaired Student’s *t*-test (Graphpad Prism V7.0d).

### Elemental bio-imaging

LA-ICP-MS experiments were performed with a CETAC LSX-213 G2+ laser ablation system (Teledyne CETAC Technologies, USA) and coupled to a Thermo iCAP RQ ICP-MS (Thermo Fisher). Helium was used as the carrier gas (99.999% purity). The LA-ICP-MS system was tuned for maximum sensitivity prior to each experiment using the reference material NIST 612 “Trace Elements in Glass”. The ICP-MS was operated in standard mode and also tuned to minimize the formation of oxides by monitoring the oxide ratio (^232^Th^16^O^+^/ ^232^Th^+^, m/z 248/232 < 0.3%). Furthermore, the isotope ratios were monitored to confirm the absence of interfering polyatomic species. Instrument parameters are summarised in S4 Table. Images were analysed and created with the imaging software MassImager 3.49 (University of Muenster, Germany). Fluorescence images were analysed with a ZEISS AxioScan Z.1 slide scanner. Each specimen was analysed under individual conditions to maximise sensitivity. Fluorescence images were normalised to optimise contrasts. External calibration using matrix matched gelatine standards was used for the quantification of Zn. Calibration curves were constructed by plotting the signal intensity of ^66^Zn^+^ obtained by LA-ICP-MS against the standards concentration (1, 5, 10, 15, 20 and 25 ppm). The correlation coefficient as a measure of linearity was determined to be 0.9992. Using the obtained linear regressions, each data point (voxel) recorded by LA-ICP-MS was converted into concentrations. The exact Zn levels of the different gelatine standards were determined in triplicate by solution nebulisation ICP-MS.

### Statistical analysis

Statistical analyses were performed with the Prism software (GraphPad Prism V7.0d). Grouped data were analysed by one- or two-way ANOVA followed by multiple comparisons (Tukey or Sidak post-hoc tests). Non-grouped analyses were performed using the Mann-Whitney test. Kaplan-Meier survival curves were analysed by Log-Rank (Mantel-Cox) test. Statistical significance was computed at P ≤ 0.5. *P ≤ 0.05; **P ≤ 0.01; ***P ≤ 0.001; and ****P ≤ 0.0001. Number of animals and replicates for each experiment are indicated in the figure legends.

## Supporting information

S1 Fig The effect of dietary Zn restriction on murine Zn abundance. The effect of dietary intervention and subsequent Zn supplementation on serum Zn levels. Outbred female Swiss mice (2 weeks old) were fed a modified diet low in Zn and their water was supplemented with 0, 100 or 250 ppm and compared to mice fed a standard laboratory rodent chow. After 2 weeks, the serum of mice (n ≥ 4) was analysed by ICP-MS. The data represent the mean (± S.E.M.) with statistical analyses performed using a one-way ANOVA.
(TIF)

S2 Fig Co-localisation of *S. pneumoniae* and Zn in murine lung tissue. Spatial distribution of Zn in the lungs of infected Zn-replete (A) or naïve Zn-replete (C) mice at 36 hrs post challenge. These data are representative elemental maps of murine tissue sections from at least two distinct murine samples by laser-ablation-ICP-MS with quantitated regions of interest (ROI) highlighted. The scale bar represents a heat map (blue to red) for the intensity of Zn from 0 to 20.0 μg.g^-1^. Spatial distribution of *S*. *pneumoniae* pVA838-GFP fluorescence in the lungs of infected Zn-replete (B) or naïve Zn-replete (D) mice at 36 hrs post challenge. The data are representative murine tissue sections from at least two distinct murine samples analysed by fluorescence microscopy with regions of interest (ROI) highlighted. The scale bar represents a heat map (black to green) for the relative fluorescence intensity.
(TIF)

S3 Fig The effect of host Zn on pneumococcal metal ion homeostasis. Proliferation of the *S*. *pneumoniae* mutant strains, Δ*adcA* and Δ*adcAII*, compared to the wild-type in the lungs (A), pleural lavage (B) and blood (C) at 36 hrs post challenge (n ≥ 5). The data represent the mean (±S.E.M.) of two independent experiments with statistical analyses performed using Student’s *t*-test. (D) The impact of Zn on Mn accumulation in *S*. *pneumoniae* was determined by *in vitro* growth in CDM supplemented with metal ions as indicated. The data represent the mean (± S.E.M.) of three independent experiments with statistical analyses performed by a one-way ANOVA with Tukey post-test. (E) The impact of 40 μM Zn relative to 12 μM Zn on transcription of *psaA*, *czcD* and *phtE* is shown. The data represent the mean (±S.E.M.) of three independent experiments with statistical analyses performed using Student’s *t*-test. Mn abundance in Zn-restricted or Zn-replete mice prior to infection (naïve) or 36 hrs post infection (n ≥ 7) in the lungs (F), pleural lavage (G) and blood serum (H). Mn concentration was determined by ICP-MS with the data representing the mean (± S.E.M.) of three independent experiments with statistical analyses performed using Student’s *t*-test. (I) Spatial distribution of Mn in the lungs of Zn-restricted or Zn-replete mice, prior to (naïve) and 36 hrs post challenge. The data are representative elemental maps (from independent tissue analyses from three distinct mice) analysed by laser-ablation-ICP-MS. The scale bar represents a heat map (blue to red) for the intensity of Mn from 0 to 0.4 μg.g^-1^.
(TIF)

S4 Fig The effect of pneumococcal infection on calprotectin expression. Calprotectin expression in Zn-restricted and Zn-replete mice prior to (naïve) or 36 hrs post infection was assessed in the lungs by (A) transcription of *S100A8* (n ≥ 6) and immunoblotting of (B) S100A8 and (C) S100A9 (n = 4). Calprotectin expression in the blood was determined by (D) *S100A8* transcription (n ≥ 6). Data represent the mean (± S.E.M.) from two (B, C) or three (A, D) independent experiments with statistical analyses performed using a one-way ANOVA.
(TIF)

S5 Fig The effect of Zn on the NF-κB response pathway during pneumococcal infection. Transcriptional response of *nfkb-1* (A) and *nfkb-2* (B) in the lungs of Zn-restricted and Zn-replete mice prior to (naïve) or 36 hrs post infection (n = 3). Data represent the mean (± S.E.M.) with statistical analyses performed using a one-way ANOVA. (C) Ratio of phosphorylated P65 (P-P65) to unphosphorylated P65 (P65), determined by immunoblotting (n = 4), in the lungs of Zn-restricted and Zn-replete mice prior to (naïve) or 36 hrs post infection. Data represent the mean (± S.E.M.) from three independent experiments with statistical analyses performed using a one-way ANOVA Transcriptional response of *nfkb-1* (D) and *nfkb-2* (E) in the blood of Zn-restricted and Zn-replete mice prior to (naïve) or 36 hrs post infection (n = 3). Data represent the mean (± S.E.M.) from three independent experiments with statistical analyses performed using a one-way ANOVA. (F) Ratio of phosphorylated P65 (P-P65) to unphosphorylated P65 (P65), determined by immunoblotting (n = 4), in the blood of Zn-restricted and Zn-replete mice prior to (naïve) or 36 hrs post infection. Data represent the mean (± S.E.M.) from three independent experiments with statistical analyses performed using a one-way ANOVA.
(TIF)

S6 Fig Gating strategies used for lung tissue analyses. (A) Lung alveolar macrophages. Cells were gated as shown to exclude debris, doublets, and dead cells (top 3 panels). Cells were further gated for CD45 expression, and live CD45^+^ cells then analysed for F4/80 and CD11b expression. F4/80^+^ CD11b^low/-^ cells were further analysed for markers expressed by alveolar macrophages, specifically Siglec F and CD11c. (B) Lung monocytes. Cells were gated as shown to exclude debris, doublets, and dead cells (top 3 panels). Cells were further gated for CD45 expression, and CD11b expression. CD45^+^ CD11b^+^ cells were then analysed for markers expressed by monocytes, specifically Gr1 and F4/80. (C) Lung neutrophils. Cells were gated as shown to exclude debris, doublets, and dead cells (top 3 panels). Cells were further gated for CD45 expression, and CD11b expression. CD45^+^ CD11b^+^ cells were then analysed for markers expressed by neutrophils, specifically high levels of Gr1 (Gr1^++^), and the absence of F4/80.
(TIF)

S7 Fig Gating strategies used for blood tissue analyses. Blood monocytes and neutrophils. Cells were gated as shown to exclude debris, doublets, and dead cells (top 3 panels). Cells were further gated for CD45 expression, and live CD45^+^ cells were then analysed for CD11b and Ly6C expression as shown. Monocytes are CD11b^+^ Ly6C^hi^ while neutrophils are CD11b^+^ Ly6C^+^ (i.e. lower expression of Ly6C than monocytes).
(TIF)

S8 Fig Gating strategy for PMN analyses. The gating strategy used to measure Zn levels in peripheral blood PMNs by flow cytometry in Fig 5A and described in the Material and Methods is shown. Cells were labelled with fluorochrome conjugated antibodies, fixable live/dead dye and Fluozin-3-AM. Gating on PMNs was achieved by gating on leukocytes within the single cell gates that were live CD45^+^CD11b^+^Gr-1^+^Ly6C^int^. A representative histogram overlay shows Fluozin-3-AM labelled PMNs from mice on zinc-restricted (red) and zinc-replete (blue) diets alongside PMNs that were not labelled with Fluozin-3-AM (‘-FLZ3’ in grey).
(TIF)

S1 Table Murine tissue transition metal ion concentration. (DOCX)

S2 Table Zinc and manganese abundance in murine tissue sections. (DOCX)

S3 Table Oligonucleotide sequences. (DOCX)

S4 Table Operating conditions for the ICP-MS and laser ablation system. (DOCX)
