# Supplementary figures and images for: Dietary zinc and the control of Streptococcus pneumoniae infection
Source: PLoS Pathog. 2019 Aug 22;15(8):e1007957. doi: 10.1371/journal.ppat.1007957 (PMC6705770; doi:10.1371/journal.ppat.1007957)

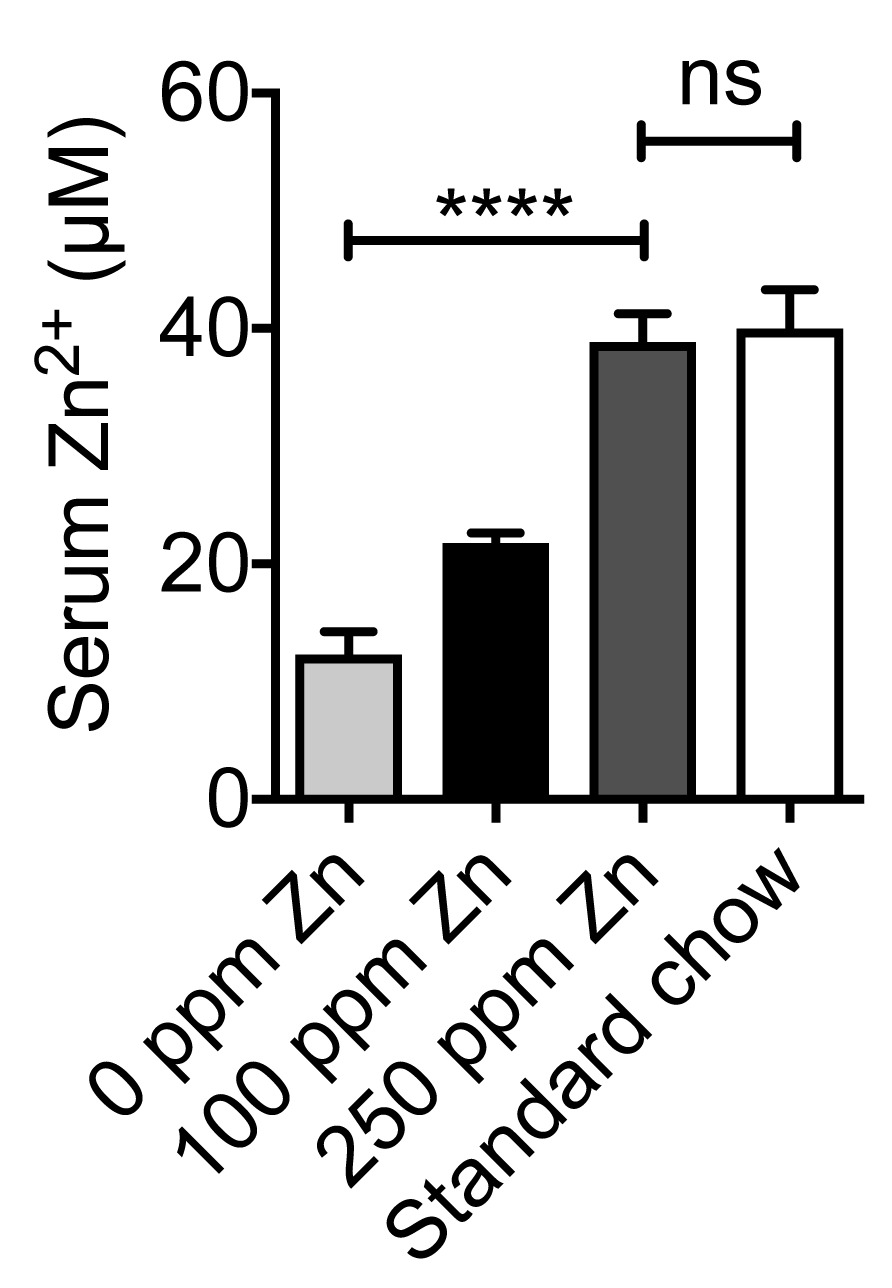

Supplement: S1 Fig — The effect of dietary intervention and subsequent Zn supplementation on serum Zn levels. Outbred female Swiss mice (2 weeks old) were fed a modified diet low in Zn and their water was supplemented with 0, 100 or 250 ppm and compared to mice fed a standard laboratory rodent chow. After 2 weeks, the serum of mice (n ≥ 4) was analysed by ICP-MS. The data represent the mean (± S.E.M.) with statistical analyses performed using a one-way ANOVA. (TIF) [file ppat.1007957.s001.tif]

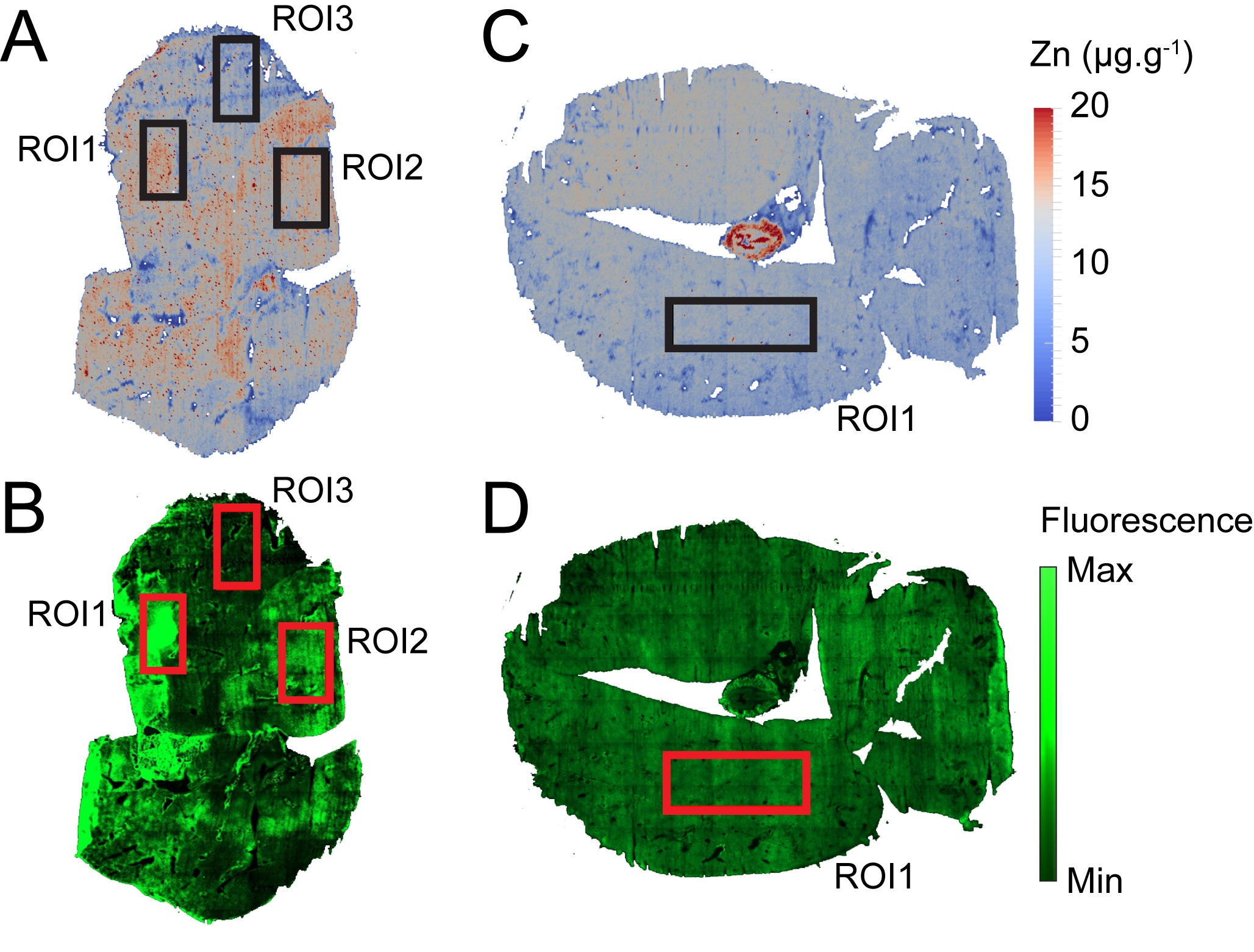

Supplement: S2 Fig — Spatial distribution of Zn in the lungs of infected Zn-replete (A) or naïve Zn-replete (C) mice at 36 hrs post challenge. These data are representative elemental maps of murine tissue sections from at least two distinct murine samples by laser-ablation-ICP-MS with quantitated regions of interest (ROI) highlighted. The scale bar represents a heat map (blue to red) for the intensity of Zn from 0 to 20.0 μg.g-1. Spatial distribution of S. pneumoniae pVA838-GFP fluorescence in the lungs of infected Zn-replete (B) or naïve Zn-replete (D) mice at 36 hrs post challenge. The data are representative murine tissue sections from at least two distinct murine samples analysed by fluorescence microscopy with regions of interest (ROI) highlighted. The scale bar represents a heat map (black to green) for the relative fluorescence intensity. (TIF) [file ppat.1007957.s002.tif]

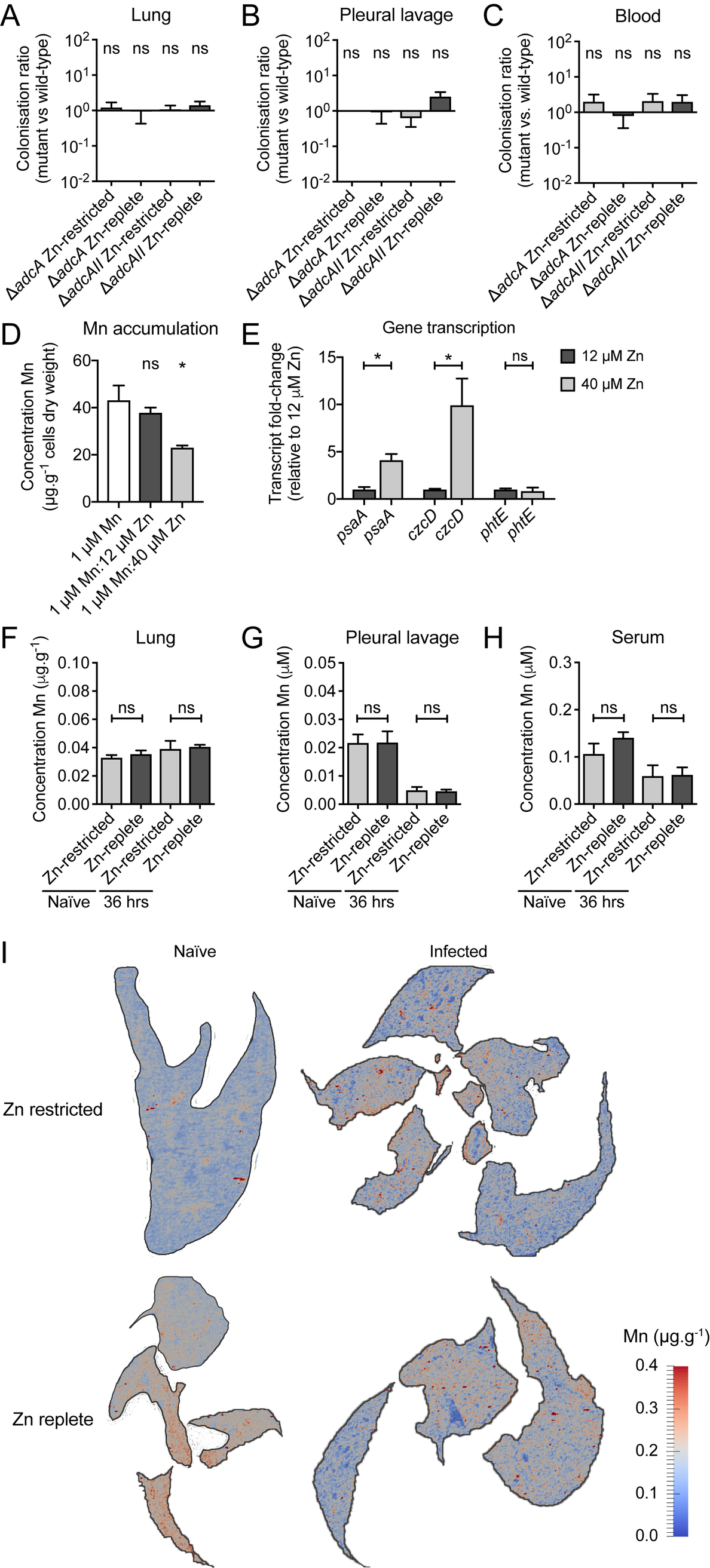

Supplement: S3 Fig — Proliferation of the S. pneumoniae mutant strains, ΔadcA and ΔadcAII, compared to the wild-type in the lungs (A), pleural lavage (B) and blood (C) at 36 hrs post challenge (n ≥ 5). The data represent the mean (±S.E.M.) of two independent experiments with statistical analyses performed using Student’s t-test. (D) The impact of Zn on Mn accumulation in S. pneumoniae was determined by in vitro growth in CDM supplemented with metal ions as indicated. The data represent the mean (± S.E.M.) of three independent experiments with statistical analyses performed by a one-way ANOVA with Tukey post-test. (E) The impact of 40 μM Zn relative to 12 μM Zn on transcription of psaA, czcD and phtE is shown. The data represent the mean (±S.E.M.) of three independent experiments with statistical analyses performed using Student’s t-test. Mn abundance in Zn-restricted or Zn-replete mice prior to infection (naïve) or 36 hrs post infection (n ≥ 7) in the lungs (F), pleural lavage (G) and blood serum (H). Mn concentration was determined by ICP-MS with the data representing the mean (± S.E.M.) of three independent experiments with statistical analyses performed using Student’s t-test. (I) Spatial distribution of Mn in the lungs of Zn-restricted or Zn-replete mice, prior to (naïve) and 36 hrs post challenge. The data are representative elemental maps (from independent tissue analyses from three distinct mice) analysed by laser-ablation-ICP-MS. The scale bar represents a heat map (blue to red) for the intensity of Mn from 0 to 0.4 μg.g-1. (TIF) [file ppat.1007957.s003.tif]

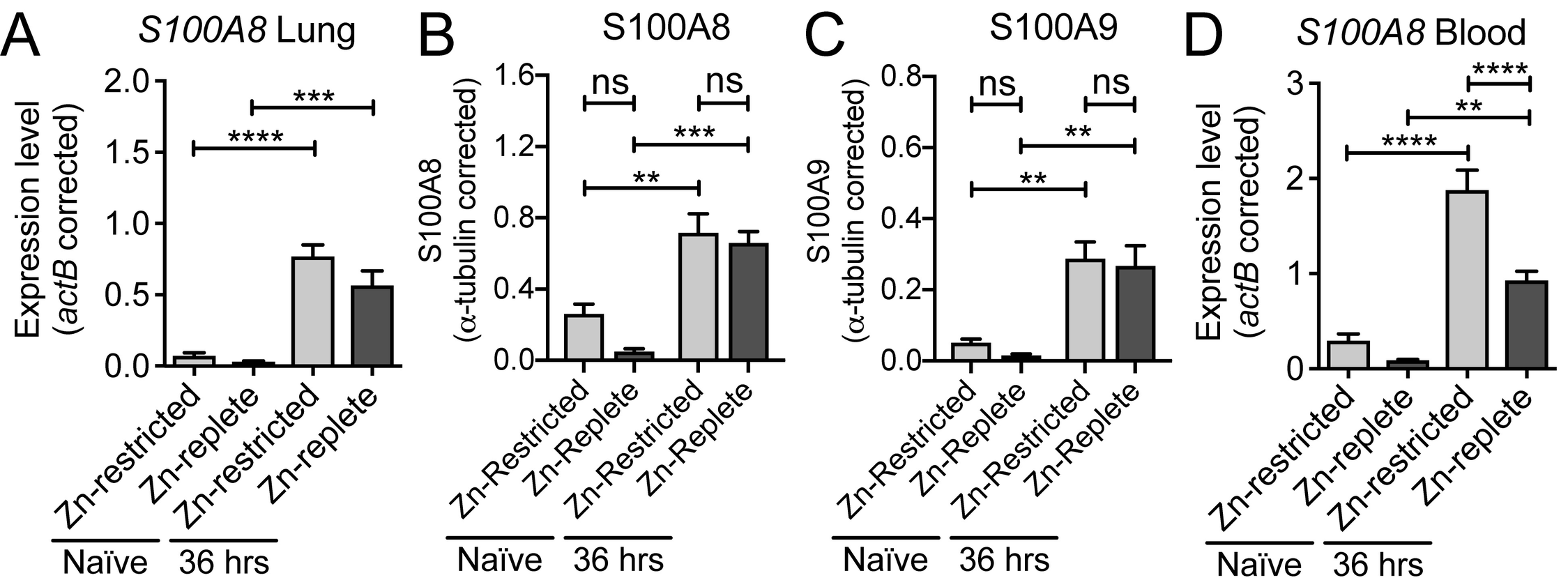

Supplement: S4 Fig — Calprotectin expression in Zn-restricted and Zn-replete mice prior to (naïve) or 36 hrs post infection was assessed in the lungs by (A) transcription of S100A8 (n ≥ 6) and immunoblotting of (B) S100A8 and (C) S100A9 (n = 4). Calprotectin expression in the blood was determined by (D) S100A8 transcription (n ≥ 6). Data represent the mean (± S.E.M.) from two (B, C) or three (A, D) independent experiments with statistical analyses performed using a one-way ANOVA. (TIF) [file ppat.1007957.s004.tif]

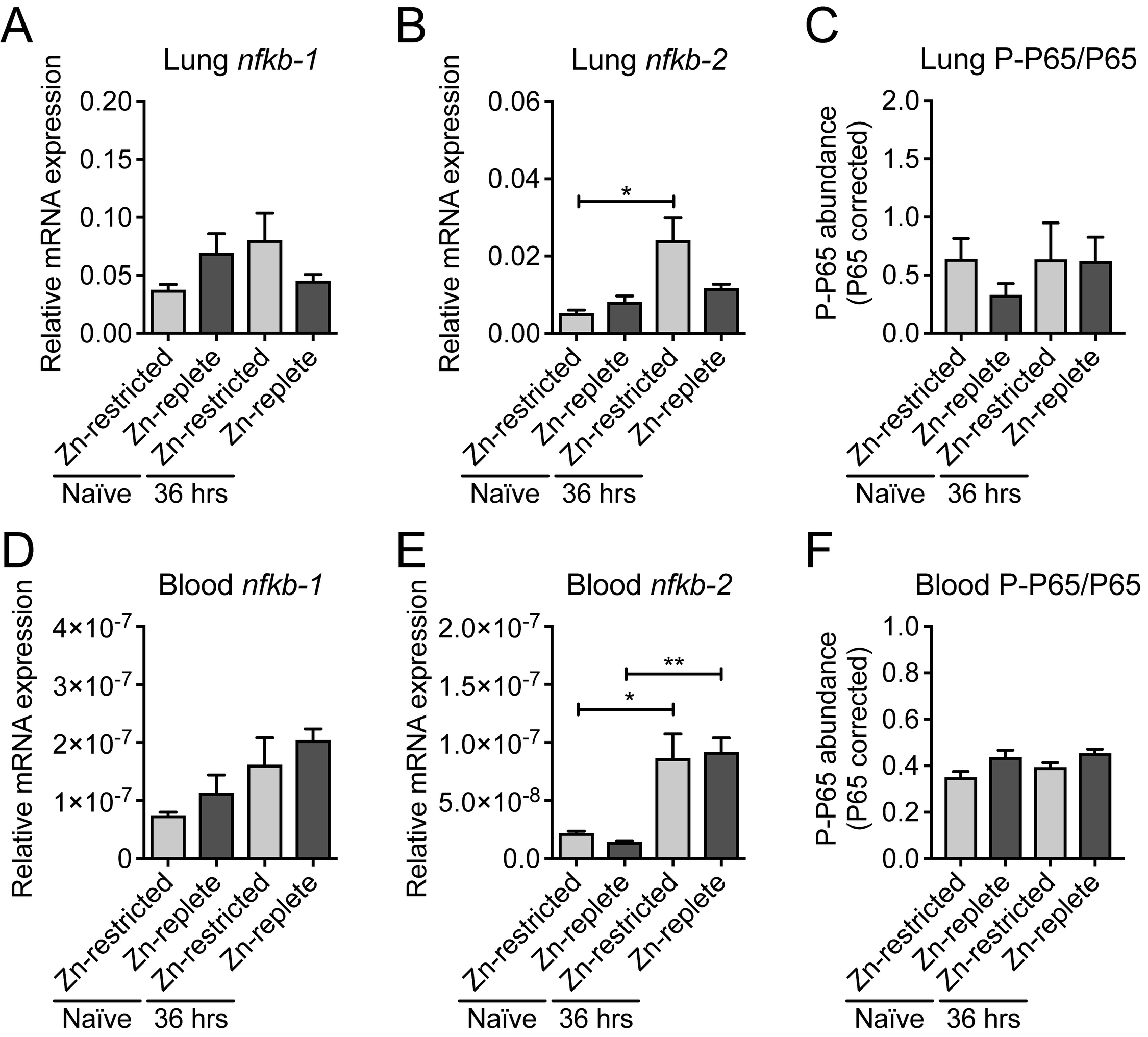

Supplement: S5 Fig — Transcriptional response of nfkb-1 (A) and nfkb-2 (B) in the lungs of Zn-restricted and Zn-replete mice prior to (naïve) or 36 hrs post infection (n = 3). Data represent the mean (± S.E.M.) with statistical analyses performed using a one-way ANOVA. (C) Ratio of phosphorylated P65 (P-P65) to unphosphorylated P65 (P65), determined by immunoblotting (n = 4), in the lungs of Zn-restricted and Zn-replete mice prior to (naïve) or 36 hrs post infection. Data represent the mean (± S.E.M.) from three independent experiments with statistical analyses performed using a one-way ANOVA Transcriptional response of nfkb-1 (D) and nfkb-2 (E) in the blood of Zn-restricted and Zn-replete mice prior to (naïve) or 36 hrs post infection (n = 3). Data represent the mean (± S.E.M.) from three independent experiments with statistical analyses performed using a one-way ANOVA. (F) Ratio of phosphorylated P65 (P-P65) to unphosphorylated P65 (P65), determined by immunoblotting (n = 4), in the blood of Zn-restricted and Zn-replete mice prior to (naïve) or 36 hrs post infection. Data represent the mean (± S.E.M.) from three independent experiments with statistical analyses performed using a one-way ANOVA. (TIF) [file ppat.1007957.s005.tif]

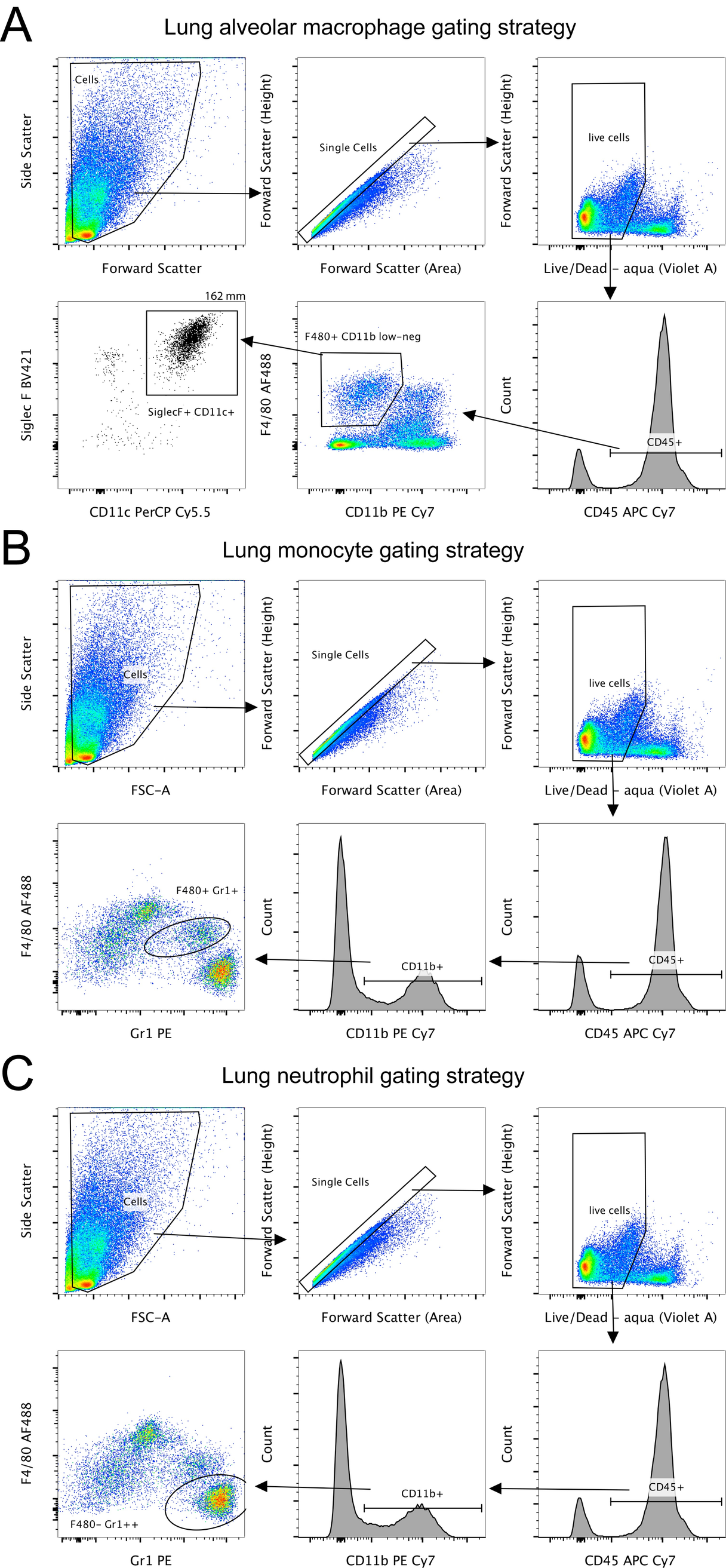

Supplement: S6 Fig — (A) Lung alveolar macrophages. Cells were gated as shown to exclude debris, doublets, and dead cells (top 3 panels). Cells were further gated for CD45 expression, and live CD45+ cells then analysed for F4/80 and CD11b expression. F4/80+ CD11blow/- cells were further analysed for markers expressed by alveolar macrophages, specifically Siglec F and CD11c. (B) Lung monocytes. Cells were gated as shown to exclude debris, doublets, and dead cells (top 3 panels). Cells were further gated for CD45 expression, and CD11b expression. CD45+ CD11b+ cells were then analysed for markers expressed by monocytes, specifically Gr1 and F4/80. (C) Lung neutrophils. Cells were gated as shown to exclude debris, doublets, and dead cells (top 3 panels). Cells were further gated for CD45 expression, and CD11b expression. CD45+ CD11b+ cells were then analysed for markers expressed by neutrophils, specifically high levels of Gr1 (Gr1++), and the absence of F4/80. (TIF) [file ppat.1007957.s006.tif]

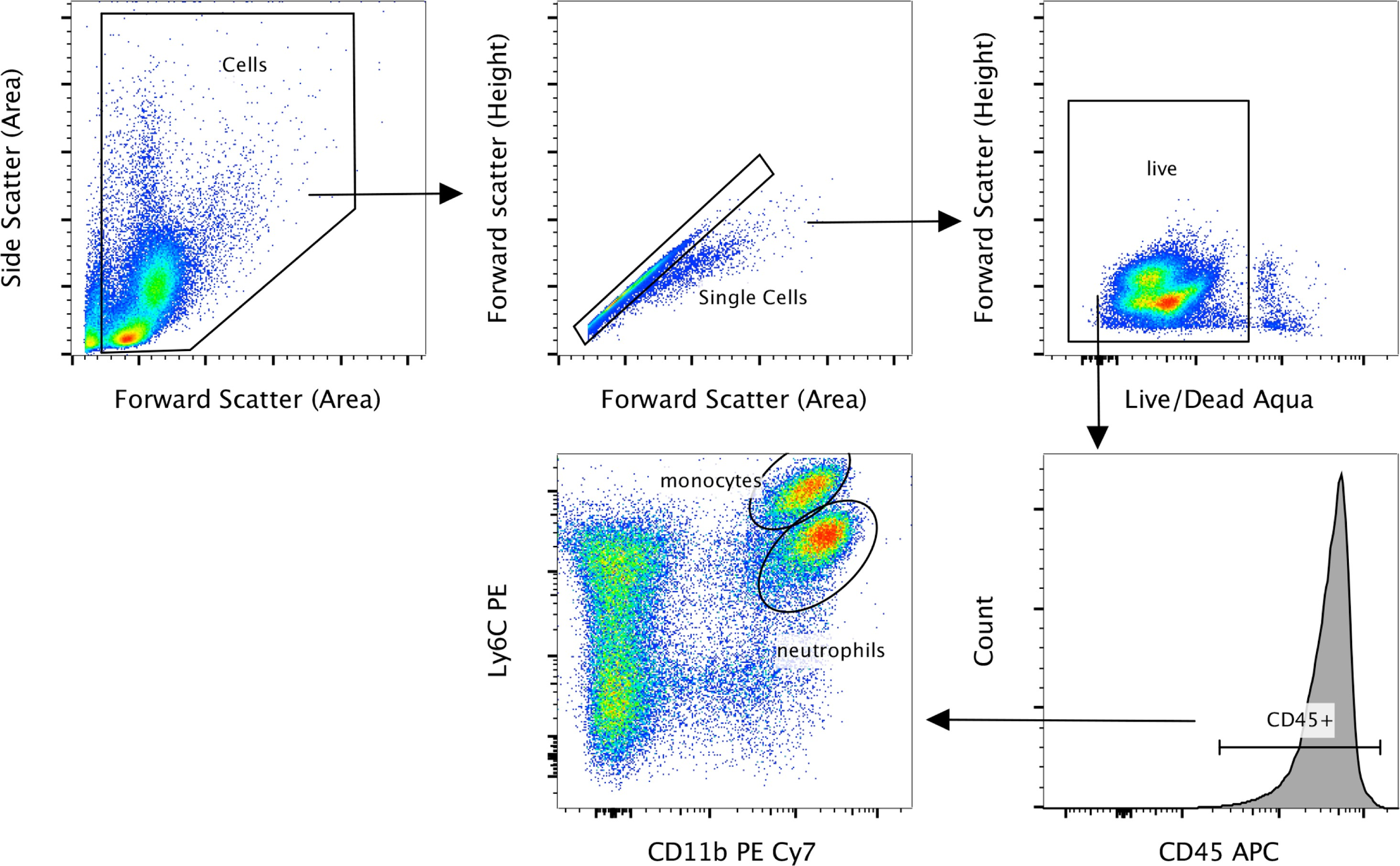

Supplement: S7 Fig — Blood monocytes and neutrophils. Cells were gated as shown to exclude debris, doublets, and dead cells (top 3 panels). Cells were further gated for CD45 expression, and live CD45+ cells were then analysed for CD11b and Ly6C expression as shown. Monocytes are CD11b+ Ly6Chi while neutrophils are CD11b+ Ly6C+ (i.e. lower expression of Ly6C than monocytes). (TIF) [file ppat.1007957.s007.tif]

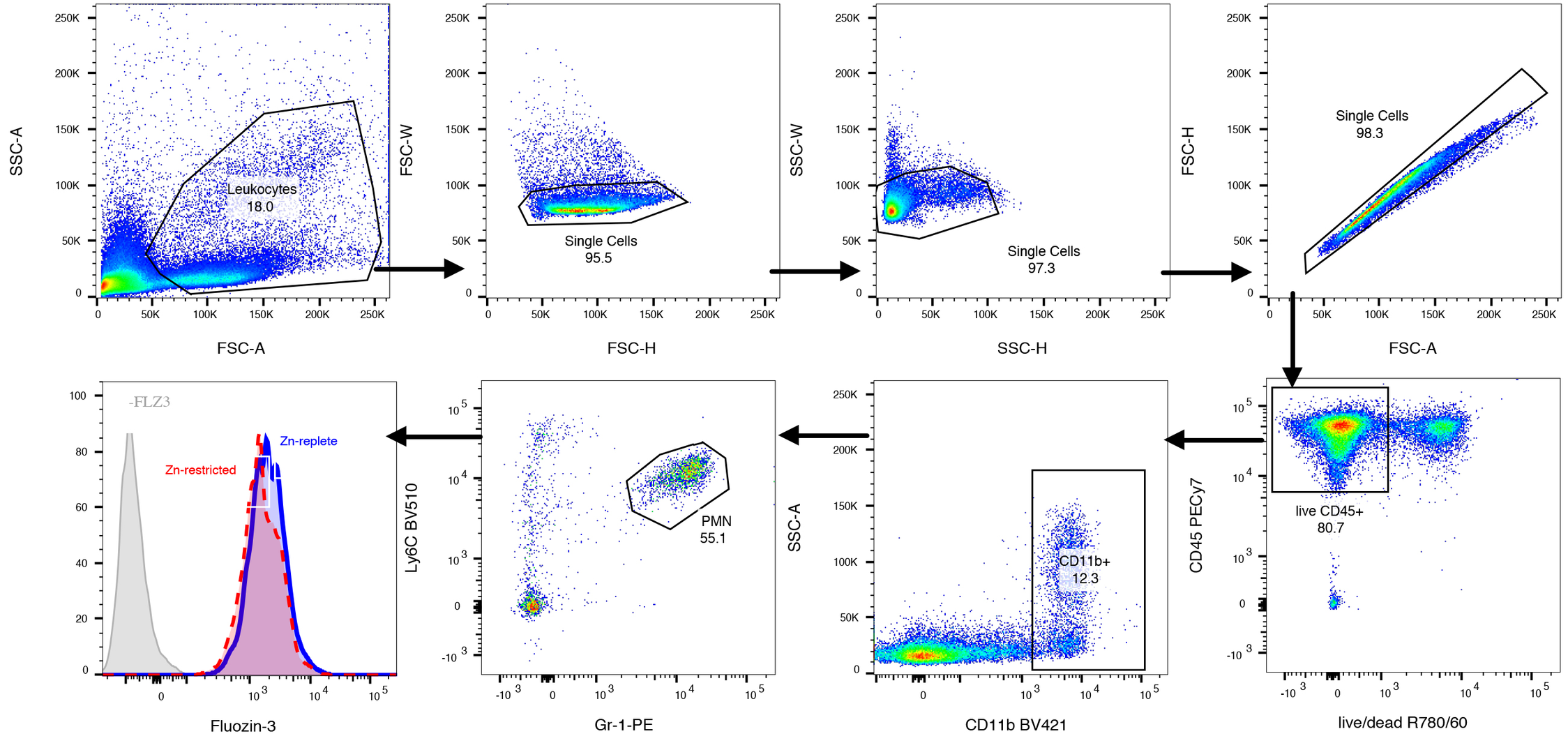

Supplement: S8 Fig — The gating strategy used to measure Zn levels in peripheral blood PMNs by flow cytometry in Fig 5A and described in the Material and Methods is shown. Cells were labelled with fluorochrome conjugated antibodies, fixable live/dead dye and Fluozin-3-AM. Gating on PMNs was achieved by gating on leukocytes within the single cell gates that were live CD45+CD11b+Gr-1+Ly6Cint. A representative histogram overlay shows Fluozin-3-AM labelled PMNs from mice on zinc-restricted (red) and zinc-replete (blue) diets alongside PMNs that were not labelled with Fluozin-3-AM (‘-FLZ3’ in grey). (TIF) [file ppat.1007957.s008.tif]
