# Supplementary material for: Dietary zinc and the control of Streptococcus pneumoniae infection
Source: PLoS Pathog. 2019 Aug 22;15(8):e1007957. doi: 10.1371/journal.ppat.1007957 (PMC6705770; doi:10.1371/journal.ppat.1007957)
Supplement: S1 Table — (DOCX) [file ppat.1007957.s009.docx]

**S1 Table. Murine tissue transition metal ion concentration**

| Metal Ion | Tissue | Naïve  (Mean concentration ± S.E.M.)^a^ | 24 hrs Post Infection (Mean concentration ± S.E.M.)^a^ | 36 hrs Post Infection (Mean concentration ± S.E.M.)^a^ |
| --- | --- | --- | --- | --- |
| Mn | Serum, Zn-restricted (μM) | 0.106 ± 0.022 | b.d. ^b^ | 0.059 ± 0.023 |
|  | Serum, Zn-replete (μM) | 0.141 ± 0.012 | b.d. ^b^ | 0.062 ± 0.016 |
|  | Pleural Lavage, Zn-restricted (μM) | 0.022 ± 0.003 | b.d. ^b^ | 0.005 ± 0.001 |
|  | Pleural Lavage, Zn-replete (μM) | 0.022 ± 0.004 | b.d. ^b^ | 0.005 ± 0.001 |
|  | Lungs, Zn-restricted (μg.g^-1^) | 0.446 ± 0.026 | 0.237 ± 0.022 | 0.545 ± 0.079 |
|  | Lungs, Zn-replete (μg.g^-1^) | 0.480 ± 0.035 | 0.429 ± 0.037 | 0.550 ± 0.020 |
|  | Nasopharynx, Zn-restricted (μg.g^-1^) | 1.011 ± 0.074 | 1.240 ± 0.094 | 0.923 ± 0.083 |
|  | Nasopharynx, Zn-replete (μg.g^-1^) | 1.518 ± 0.138 | 2.631 ± 0.375 | 1.607 ± 0.192 |
| Fe | Serum, Zn-restricted (μM) | 78.35 ± 5.43 | 36.14 ± 6.086 | 39.55 ± 6.335 |
|  | Serum, Zn-replete (μM) | 60.14 ± 4.90 | 26.20 ± 2.464 | 36.56 ± 8.362 |
|  | Pleural Lavage, Zn-restricted (μM) | 1.123 ± 0.181 | 1.151 ± 0.165 | 1.386 ± 0.248 |
|  | Pleural Lavage, Zn-replete (μM) | 1.007 ± 0.199 | 0.904 ± 0.232 | 1.547 ± 0.346 |
|  | Lungs, Zn-restricted (μg.g^-1^) | 169.20 ± 15.24 | 80.92 ± 14.35 | 144.10 ± 9.17 |
|  | Lungs, Zn-replete (μg.g^-1^) | 152.20 ± 17.56 | 111.10 ± 9.81 | 142.60 ± 9.68 |
|  | Nasopharynx, Zn-restricted (μg.g^-1^) | 232.30 ± 24.77 | 430.60 ± 34.86 | 336.10 ± 31.64 |
|  | Nasopharynx, Zn-replete (μg.g^-1^) | 215.60 ± 29.05 | 917.20 ± 85.25 | 354.10 ± 37.66 |
| Co | Serum, Zn-restricted (μM) | 0.030 ± 0.004 | 0.021 ± 0.002 | 0.034 ± 0.005 |
|  | Serum, Zn-replete (μM) | 0.030 ± 0.003 | 0.033 ± 0.002 | 0.046 ± 0.011 |
|  | Pleural Lavage, Zn-restricted (μM) | 0.002 ± 0.000 | 0.002 ± 0.000 | 0.002 ± 0.000 |
|  | Pleural Lavage, Zn-replete (μM) | 0.002 ± 0.000 | 0.002 ± 0.000 | 0.003 ± 0.000 |
|  | Lungs, Zn-restricted (μg.g^-1^) | 0.016 ± 0.002 | 0.015 ± 0.005 | 0.010 ± 0.001 |
|  | Lungs, Zn-replete (μg.g^-1^) | 0.018 ± 0.004 | 0.010 ± 0.001 | 0.015 ± 0.003 |
|  | Nasopharynx, Zn-restricted (μg.g^-1^) | 0.055 ± 0.004 | 0.082 ± 0.007 | 0.063 ± 0.007 |
|  | Nasopharynx, Zn-replete (μg.g^-1^) | 0.066 ± 0.006 | 0.305 ± 0.024 | 0.070 ± 0.007 |
| Ni | Serum, Zn-restricted (μM) | 0.232 ± 0.046 | 2.047 ± 0.481 | 2.730 ± 0.421 |
|  | Serum, Zn-replete (μM) | 0.178 ± 0.049 | 1.301 ± 0.026 | 3.217 ± 0.777 |
|  | Pleural Lavage, Zn-restricted (μM) | 0.044 ± 0.011 | 0.177 ± 0.004 | 0.241 ± 0.010 |
|  | Pleural Lavage, Zn-replete (μM) | 0.058 ± 0.020 | 0.177 ± 0.004 | 0.240 ± 0.007 |
|  | Lungs, Zn-restricted (μg.g^-1^) | 0.208 ± 0.049 | 1.202 ± 0.280 | 0.756 ± 0.124 |
|  | Lungs, Zn-replete (μg.g^-1^) | 0.247 ± 0.063 | 0.767 ± 0.120 | 0.821 ± 0.123 |
|  | Nasopharynx, Zn-restricted (μg.g^-1^) | 0.450 ± 0.043 | 0.605 ± 0.050 | 1.193 ± 0.149 |
|  | Nasopharynx, Zn-replete (μg.g^-1^) | 0.486 ± 0.025 | 1.946 ± 0.043 | 1.247 ± 0.149 |
| Cu | Serum, Zn-restricted (μM) | 3.257 ± 0.978 | 18.27 ± 1.166 | 32.11 ± 4.700 |
|  | Serum, Zn-replete (μM) | 4.622 ± 0.682 | 18.16 ± 0.683 | 34.69 ± 6.198 |
|  | Pleural Lavage, Zn-restricted (μM) | 3.940 ± 0.353 | 1.382 ± 0.092 | 3.168 ± 0.215 |
|  | Pleural Lavage, Zn-replete (μM) | 6.667 ± 1.125 | 2.080 ± 0.172 | 4.379 ± 0.368 |
|  | Lungs, Zn-restricted (μg.g^-1^) | 1.349 ± 0.242 | 9.960 ± 1.081 | 20.67 ± 1.682 |
|  | Lungs, Zn-replete (μg.g^-1^) | 1.093 ± 0.287 | 12.30 ± 2.496 | 16.92 ± 1.424 |
|  | Nasopharynx, Zn-restricted (μg.g^-1^) | 5.889 ± 0.414 | 13.19 ± 2.307 | 7.333 ± 1.122 |
|  | Nasopharynx, Zn-replete (μg.g^-1^) | 7.525 ± 0.885 | 10.33 ± 0.141 | 7.626 ± 1.042 |
| Zn | Serum, Zn-restricted (μM) | 12.31 ± 1.923 | 10.08 ± 1.533 | 8.334 ± 2.586 |
|  | Serum, Zn-replete (μM) | 38.84 ± 2.418 | 21.94 ± 3.768 | 28.79 ± 3.513 |
|  | Pleural Lavage, Zn-restricted (μM) | 3.169 ± 0.346 | 1.441 ± 0.039 | 2.492 ± 0.507 |
|  | Pleural Lavage, Zn-replete (μM) | 3.528 ± 0.281 | 1.616 ± 0.039 | 2.804 ± 0.514 |
|  | Lungs, Zn-restricted (μg.g^-1^) | 166.20 ± 10.22 | 221.0 ± 10.47 | 198.90 ± 9.85 |
|  | Lungs, Zn-replete (μg.g^-1^) | 179.0 ± 8.84 | 225.60 ± 11.56 | 244.10 ± 11.96 |
|  | Nasopharynx, Zn-restricted (μg.g^-1^) | 451.60 ± 33.01 | 595.90 ± 34.42 | 446.30 ± 38.20 |
|  | Nasopharynx, Zn-replete (μg.g^-1^) | 1667.0 ± 124.40 | 2078.0 ± 336.6 | 1789.0 ± 155.8 |

1. Data represent the mean (± S.E.M.) of four independent experiments.
2. Below detection (b.d.)
