# Supplementary material for: Dietary zinc and the control of Streptococcus pneumoniae infection
Source: PLoS Pathog. 2019 Aug 22;15(8):e1007957. doi: 10.1371/journal.ppat.1007957 (PMC6705770; doi:10.1371/journal.ppat.1007957)
Supplement: S2 Table — (DOCX) [file ppat.1007957.s010.docx]

**S2 Table. Zinc and manganese abundance in murine tissue sections.**

|  | Tissue Sections (μg metal.g^-1^) ^a^ | | | |
| --- | --- | --- | --- | --- |
|  | Replete Naïve | Restricted Naïve | Replete Infected | Restricted Infected |
| Zinc | 7.86 ± 1.08 | 1.35 ± 0.53 | 9.55 ± 1.21 | 2.56 ± 0.25 |
| Manganese | 0.17 ± 0.01 | 0.21 ± 0.01 | 0.19 ± 0.01 | 0.24 ± 0.02 |

1. Data represent the mean (± S.E.M.) of at least two (n ≥ 2) independent murine tissue section analyses.
