# Supplementary material for: Dietary zinc and the control of Streptococcus pneumoniae infection
Source: PLoS Pathog. 2019 Aug 22;15(8):e1007957. doi: 10.1371/journal.ppat.1007957 (PMC6705770; doi:10.1371/journal.ppat.1007957)
Supplement: S3 Table — (DOCX) [file ppat.1007957.s011.docx]

**S3 Table. Oligonucleotide sequences**

| Organism | Name | Forward (5’ → 3’) | Reverse (5’ → 3’) | Reference |
| --- | --- | --- | --- | --- |
| D39 | 16s | CATGCAAGTAGAACGCTGAA | TGTCATGCAACATCCACTCT | This study |
| D39 | *psaA* | AGCCTATGGTGTTCCAAGTG | GTTTTCATTGGACGGTCATC | This study |
| D39 | *czcD* | TTGGTTCTAGCGCTGTTCTT | AGGCTCCTAGCAGGCTAAAC | This study |
| D39 | *phtE* | GGAACAGTTGAGAACCAACCA | TAGGGTCACTCCCCACATTC | This study |
| *Mus musculus* | S100A8 | CCTTTGTCAGCTCCGTCTTC | TAGAGGGCATGGTGATTTCC | This study |
| *Mus musculus* | Actb | GGCTGTATTCCCCTCCATCG | CCAGTTGGTAACAATGCCATGT | PrimerBank |
| *Mus musculus* | S100A9 | TCAGACAAATGGTGGAAGCA | TCAACTTTGCCATCAGCATC | This study |
