# Supplementary material for: Dietary zinc and the control of Streptococcus pneumoniae infection
Source: PLoS Pathog. 2019 Aug 22;15(8):e1007957. doi: 10.1371/journal.ppat.1007957 (PMC6705770; doi:10.1371/journal.ppat.1007957)
Supplement: S4 Table — (DOCX) [file ppat.1007957.s012.docx]

**S4 Table. Operating conditions for the ICP-MS and laser ablation system**

| Instrument | Parameters |
| --- | --- |
| *Thermo ICAP RQ* |  |
| RF Power | 1550 W |
| Ar make-up gas | 0.9 L.min^-1^ |
| Isotopes | P^31^, Mn^55^, Fe^57^, Cu^63^ and ^66^Zn |
| Integration time | 50 ms |
| *CETAC LSX-213 G2+* |  |
| Laser wavelength | 213 nm |
| Pulse energy | 20% (1.64 J.cm^2^) |
| Repetition Rate | 20 Hz |
| Scan speed | 120 µm s^-1^ |
| Carrier gas flow rate | 1.2 L min^-1^ He |
